# Automated diagnosis of keratitis from low-quality slit-lamp images using an improved generative adversarial network

**DOI:** 10.1038/s41746-026-02952-4

**Published:** 2026-07-03

**Authors:** Jiewei Jiang, Siyuan Song, Qihai Yao, Kuan Li, Tongtong Lin, Yu Xin, Yiwei Cui, Ning Wang, Yifan Bai, Xiaoci Luo, Wei Chen, Mingmin Zhu, Lang He, Zhongwen Li

**Affiliations:** 1 School of Electronic Engineering, Xi’an University of Posts and Telecommunications, Xi’an, China; 2 Ningbo Key Laboratory of Medical Research on Blinding Eye Diseases, Ningbo Eye Institute, Ningbo Eye Hospital, Wenzhou Medical University, Ningbo, China; 3 School of Ophthalmology and Optometry and Eye Hospital, Wenzhou Medical University, Wenzhou, China; 4 School of Mathematics and Statistics, Xidian University, Xi’an, China; 5 School of Computer Science and Technology, Xi’an University of Posts and Telecommunications, Xi’an, China

**Keywords:** Computational biology and bioinformatics, Diseases, Health care, Mathematics and computing, Medical research

## Abstract

Keratitis is a major cause of visual impairment worldwide, with timely and accurate diagnosis being critical to preventing irreversible vision loss. Existing deep learning (DL) models rely heavily on high-quality slit-lamp (HQS) images, limiting their applicability in real-world clinical scenarios where low-quality slit-lamp (LQS) images are frequently encountered due to acquisition-related artifacts. Here, we propose an automated keratitis diagnosis framework for LQS images (AKDF-LQSI) that integrates image enhancement with lesion-aware classification. A random degradation algorithm is designed to simulate common clinical artifacts, enabling paired LQS–HQS training. The framework incorporates an improved generative adversarial network to restore diagnostic features and a context-aware classifier to enhance lesion representation. AKDF-LQSI demonstrates robust performance when evaluated on a multicenter dataset of 10,498 slit-lamp images, achieving an AUC exceeding 0.949. This approach presents a practical solution for keratitis diagnosis from degraded images, with strong potential to reduce diagnostic disparities in resource-limited environments.

## Introduction

‌Keratitis‌, a sight-threatening inflammatory disorder of the ocular surface, ranks the ‌fifth-leading cause of visual impairment globally, with 1.5 to 2.0 million annual cases of monocular blindness‌ representing a critical public health challenge in ocular therapeutics^1,2^. Timely and accurate diagnosis of keratitis remains paramount, as early intervention can effectively prevent severe vision impairment and, in many cases, irreversible blindness^3,4^. Nevertheless, ‌geospatial disparities in ophthalmologic care infrastructure‌ create significant diagnostic bottlenecks, particularly in resource-limited rural and remote regions where‌ potential keratitis patients lack access to timely slit-lamp diagnosis and appropriate care^5,6^.

The transformative application of artificial intelligence (AI) has fundamentally revolutionized diagnostic paradigms for various medical image–based disease recognition tasks, including keratitis^7–9^, cataracts^10–12^, ocular tumors^13–15^, diabetic retinopathy^16–19^, glaucoma^20–22^, age-related macular degeneration^23,24^, and breast cancer^25–27^. This progress has been driven by the rapid expansion of medical imaging data and advancements in machine learning^28,29^ and deep learning (DL)^30–33^ methodologies. Notably, DL-based approaches enable the systematic extraction of discriminative features and latent patterns from large-scale slit-lamp imaging datasets, thereby markedly improving the diagnostic objectivity and accuracy for keratitis^7–9,31,32^. For instance, Li et al. demonstrated that DenseNet121^34^ achieves an area under the curve (AUC) of 0.96 in differentiating keratitis from normal cornea and other cornea abnormalities using 6567 annotated slit-lamp images, with validation through ‌multicenter cohort studies‌ adhering to clinical AI verification protocols^8^. Recent innovations in fully convolutional networks with class-aware attention mechanisms further addressed subtype classification challenges, achieving 92% accuracy in distinguishing bacterial, fungal, and viral keratitis^35^.‌ Teeyapan et al. corroborated this trend, showing ResNet50 attains 95.1% accuracy in staging corneal ulcers, with performance metrics exceeding traditional diagnostic thresholds^36^. These advancements highlight the impressive potential of DL algorithms in the automatic diagnosis of keratitis.

Existing research on automated keratitis diagnosis predominantly relies on high-quality slit-lamp (HQS) images, a dependency that limits its clinical applicability in resource-constrained settings^7–9,37–40^. In real-world practice, low-quality slit-lamp (LQS) images—resulting from suboptimal acquisition techniques, patient non-cooperation, or environmental interference—are frequently encountered, particularly in regions with limited ophthalmic expertise^37–40^. Current diagnostic workflows often exclude LQS images during pre-screening, inadvertently restricting the generalizability of trained DL models to these heterogeneous LQS datasets. Preliminary studies have demonstrated that DL models achieve high performance with HQS images for keratitis diagnosis in controlled environments (AUCs >0.96), yet their effectiveness declines markedly when applied to LQS images due to domain shift and feature degradation^38,39^. This discrepancy exacerbates diagnostic inaccuracies, increases misdiagnosis risks, and imposes additional burdens on healthcare systems through repeated examinations. Efforts to enhance model robustness by incorporating LQS images into training datasets have revealed a critical trade-off: while generalization to LQS images improves, this comes at the expense of severe performance deterioration on HQS benchmark evaluations^38^. Despite this challenge, automated enhancement techniques for LQS images remain underexplored. To address this gap, we propose an image enhancement framework leveraging an innovative generative adversarial network (GAN) to map LQS images to the HQS domain, thereby improving the robustness and reliability of automated keratitis diagnosis across diverse clinical settings.

In summary, the main contributions of this study can be outlined as follows. First, we propose an automated keratitis diagnosis framework for low-quality slit-lamp images (AKDF-LQSI), designed to address the challenges posed by LQS data. Second, we develop a random degradation algorithm applied to HQS images, simulating common clinical artifacts such as motion blur (MB), defocus blur (DB), and exposure distortion (ED). These degraded images are paired with their HQS counterparts to train the GAN model. Third, we present a novel GAN architecture, termed SCUNet, which embeds a Swin-Conv module into the U-Net. By combining an adversarial learning strategy with multi-resolution feature maps, SCUNet significantly enhances the fidelity of LQS image reconstruction. Fourth, we propose the GCT-DenseNet121 model for keratitis classification, which combines a Gaussian context transformer (GCT) with DenseNet121. The GCT module captures global contextual information from slit-lamp images, effectively encoding the complex morphological features of keratitis. Finally, extensive quantitative experiments and qualitative analyses demonstrate the superior performance of the AKDF-LQSI in addressing LQS images, markedly improving the generalizability of keratitis diagnosis.

## Results

### Experimental environment

All experiments were conducted using the PyTorch deep learning framework. The SCUNet model for LQS image enhancement was trained on four NVIDIA TITAN RTX GPUs for 120 epochs with a batch size of 4. The Adam optimizer was employed with a learning rate of 3e-4, β1 of 0.5, and β2 of 0.999. Input images were uniformly resized to 512×512 pixels, and data loading was parallelized across eight CPU threads. The GCT-DenseNet121 model was optimized for 80 epochs with a batch size of 64, using mini-batch gradient descent with an initial learning rate of 0.005, β1 of 0.9, β2 of 0.999, and a weight decay of 5e-5. The network was initialized with ImageNet pre-trained weights. All slit-lamp image were resized to 224×224 pixels and normalized to the range [0,1]. To enhance data diversity and reduce overfitting and bias, data augmentation techniques were applied during training, including random cropping, horizontal and vertical flipping, and random rotation around the image center. Detailed hyperparameter configurations are provided in Supplementary Table 1. After training, we further quantified trainable parameters, training time, and average inference time per image for both SCUNet and GCT-DenseNet121, as summarized in Supplementary Table 2.

### Evaluation metrics and statistical analysis

The evaluation metrics for the classification models include accuracy, specificity, and sensitivity, as defined in Eqs. (1–5). The receiver operating characteristic (ROC) curve visualizes the model’s performance across various decision thresholds by plotting the false positive rate (FPR) on the x-axis and the true positive rate (TPR) on the y-axis, as described in Eqs. (4, 5).

1 $$Accuracy=\frac{TP+TN}{TP+FP+TN+FN}$$

2 $$Specificity=\frac{TN}{TN+FP}$$

3 $$Sensitivity=\frac{TP}{TP+FN}$$

4 $$FPR=\frac{FP}{FP+TN}$$

5 $$TPR=\frac{TP}{TP+FN}$$

where TP, FP, TN, and FN denote the number of true positive, false positive, true negative, and false negative samples, respectively. To quantitatively assess the statistical reliability of model performance, two-sided 95% confidence intervals (CIs) were calculated. Specifically, the 95% CIs for accuracy, sensitivity, and specificity were determined using the Wilson Score method implemented in the Statsmodels package (version 0.11.1), while the 95% CI for AUC was obtained via an empirical bootstrap procedure with 1000 random resamples. In human–AI comparisons, the McNemar test was performed to evaluate differences in accuracy, sensitivity, and specificity between the model and ophthalmologists of varying experience levels, with statistical significance defined as a two-sided *P* value below 0.05.

Both no-reference and reference quality assessment metrics^41^ were applied in this study to evaluate the performance of generative models. For no-reference metrics, original HQS images were not required for comparison. The mean pixel value quantifies the overall luminance of the image, the standard deviation (SD) measures the dispersion of pixel intensities, and the average gradient (AG) characterizes the contrast and sharpness of fine details within the image, as shown in Eqs. (6–8).

6 $$Mean=\frac{1}{N}\mathop{\sum }\limits_{i=1}^{N}{x}_{i}$$

7 $$SD=\sqrt{\frac{1}{N}\mathop{\sum }\limits_{i=1}^{N}{({x}_{i}-Mean)}^{2}}$$

8 $$AG=\frac{1}{N}\mathop{\sum }\limits_{i=1}^{N}\sqrt{({G}_{x}^{2}+{G}_{y}^{2})2}$$

where *N* denotes the total number of pixels, *x*_*i*_ represents the value of the *i*th pixel in the image, and *G*_*x*_ and *G*_*y*_ refer to the horizontal and vertical gradients, respectively.

Reference-based metrics require original HQS images for comparison, including mean squared error (MSE), peak signal-to-noise ratio (PSNR), and structural similarity index measure (SSIM), as defined in Eqs. (9–11). MSE calculates the average squared differences between the generated and original images, with lower values indicating a closer resemblance between the generated image and its original counterpart. PSNR, expressed in decibels (dB), is derived from MSE and provides a logarithmic measure of image fidelity. Higher PSNR values indicate smaller discrepancies between the generated and original images. SSIM offers a more comprehensive assessment by evaluating three key perceptual components: luminance, contrast, and structural integrity. It measures the structural similarity between the original and generated images, with values ranging from −1 to 1. An SSIM value of 1 signifies an identical match between the two images, whereas values approaching −1 indicate substantial structural deviations.

9 $$MSE=\frac{1}{N}\mathop{\sum }\limits_{i=1}^{N}{(I(i)-K(i))}^{2}$$

10 $$PSNR=10\cdot {\log }_{10}\left(\frac{MA{X}^{2}}{MSE}\right)$$

11 $$SSIM(x,y)=\frac{(2{\mu }_{x}{\mu }_{y}+{C}_{1})(2{\sigma }_{xy}+{C}_{2})}{({\mu }_{x}^{2}+{\mu }_{y}^{2}+{C}_{1})({\sigma }_{x}^{2}+{\sigma }_{y}^{2}+{C}_{2})}$$

where *N* and MAX represent the total number of pixels in the image and the maximum pixel intensity, respectively. *I*(*i*) and *K*(*i*) denote the intensity values of the *i*th pixel in the original and generated images, respectively. *μ*_*x*_ and *μ*_*y*_ denote average luminance values of the original and generated images, respectively, while *σ*_*x*_ and *σ*_*y*_ represent their corresponding standard deviations. *σ*_*xy*_ represents the covariance of the luminance values between the original and generated images. The constants *C*_*1*_ and *C*_*2*_ are introduced to enhance numerical stability in SSIM computations.

### Performance evaluation of SCUNet on generated LQS images in the internal test set

The trained SCUNet model was evaluated on generated low-quality slit-lamp (GLQS) images from the internal test set of Ningbo Eye Hospital (NEH). SCUNet was applied to GLQS images to obtain enhanced slit-lamp (ES) images, which were quantitatively compared with the corresponding HQS images to assess enhancement effectiveness. Both no-reference and reference metrics were calculated for three types of GLQS images: MB, DB, and ED. The average values of evaluation metrics for all test images in each category are summarized in Table 1.

**Table 1:** Performance evaluation of SCUNet on three types of GLQS images in the internal test set

Metrics	GLQS images			ES images		
	MB	DB	ED	MB	DB	ED
Mean	95.83	101.72	107.92	96.06	92.02	92.81
SD	57.44	61.56	65.44	55.83	52.67	51.24
AG	10.40	14.42	22.98	7.80	15.94	18.99
MSE	75.60	55.85	100.15	67.40	45.79	73.64
PSNR	20.48	25.66	18.41	21.82	29.71	25.73
SSIM	0.71	0.80	0.76	0.78	0.88	0.94

*SCUNet* U-net with Swin-Conv module. *GLQS* generated low-quality slit-lamp, *ES* enhanced slit-lamp, *MB* motion blur, DB defocus blur, *ED* exposure distortion. *SD* standard deviation, *AG* average gradient. *MSE* mean squared error. *PSNR* peak signal-to-noise ratio, *SSIM* structural similarity index measure.

Comparisons between GLQS and ES images reveal a general decline in most no-reference metrics, suggesting improved luminance uniformity and reduced pixel dispersion. Specifically, for MB images, the mean value increased slightly from 95.83 (GLQS) to 96.06 (ES), while SD and AG decreased from 57.44 and 10.40 to 55.83 and 7.80, respectively. This indicates enhanced luminance consistency, reduced noise, and a smoothing effect on edges and fine details, thereby alleviating the negative impact of the MB factor on image recognition. For DB images, the AG metric increased from 14.42 (GLQS) to 15.94 (ES), whereas the mean and SD decreased from 101.72 and 61.56 to 92.02 and 52.67, respectively. This demonstrates an improvement in the edges and fine details clarity of images, alongside a reduction in overall luminance variations and noise levels. For ED images, the mean, SD, and AG of ES images decrease to 92.81, 51.24, and 18.99, respectively. This suggests a reduction in overall luminance, noise, and contrast, which facilitates the recovery of image details lost due to the ED factor.

The reference-based evaluation further confirms the SCUNet model’s ability to restore image quality, as evidenced by improvements in MSE, PSNR, and SSIM. A reduction in MSE values across all three GLQS types (MB, DB, and ED), from 75.60, 55.85, and 100.15 to 67.40, 45.79, and 73.64, respectively, indicates that the ES images more closely resemble their pre-degradation HQS counterparts, exhibiting reduced pixel-wise discrepancies. The increase in PSNR values, from 20.48, 25.66, and 18.41 to 21.82, 29.71, and 25.73, respectively, reflects an improved signal-to-noise ratio, signifying noise reduction in ES images. Furthermore, the SSIM scores exhibit a notable upward trend, from 0.71, 0.80, and 0.76 to 0.78, 0.88, and 0.94, respectively, demonstrating that the ES images are structurally more consistent with the HQS images, with superior structural fidelity.

These experimental results highlight the superior performance of the SCUNet model in restoring GLQS images, significantly alleviating the discrepancies between the ES images and the original HQS images. The consistent improvements in both no-reference and reference-based metrics suggest that the SCUNet effectively learns the mapping from degraded GLQS images to HQS images, reinforcing its potential for enhancing slit-lamp imaging quality in clinical applications.

### Performance evaluation of SCUNet on GLQS images in the external test set

To assess the generalization ability of the SCUNet model, its performance was evaluated on GLQS images in an external test set derived from Zhejiang Eye Hospital (ZEH), Jiangdong Eye Hospital (JEH), and Ningbo Ophthalmic Center (NOC). These GLQS images were generated using the same degradation algorithm applied during training. The experimental results are presented in Supplementary Table 3. All no-reference evaluation metrics (Mean, SD, and AG) for the ES images enhanced by SCUNet showed a consistent downward trend when compared to the internal GLQS images, indicating a reduction in noise levels and a notable improvement in image quality. The MSE reference metric also exhibited a decreasing trend across all three types of external GLQS images, while both PSNR and SSIM showed an upward trend. These improvements suggest that the ES images are closer to their external HQS counterparts. The trends observed in the external test set were consistent with those in the internal test set, further validating the robust generalization capabilities of SCUNet in improving image quality.

### Qualitative evaluation of SCUNet on the GLQS and LQS images

**Fig. 1:**
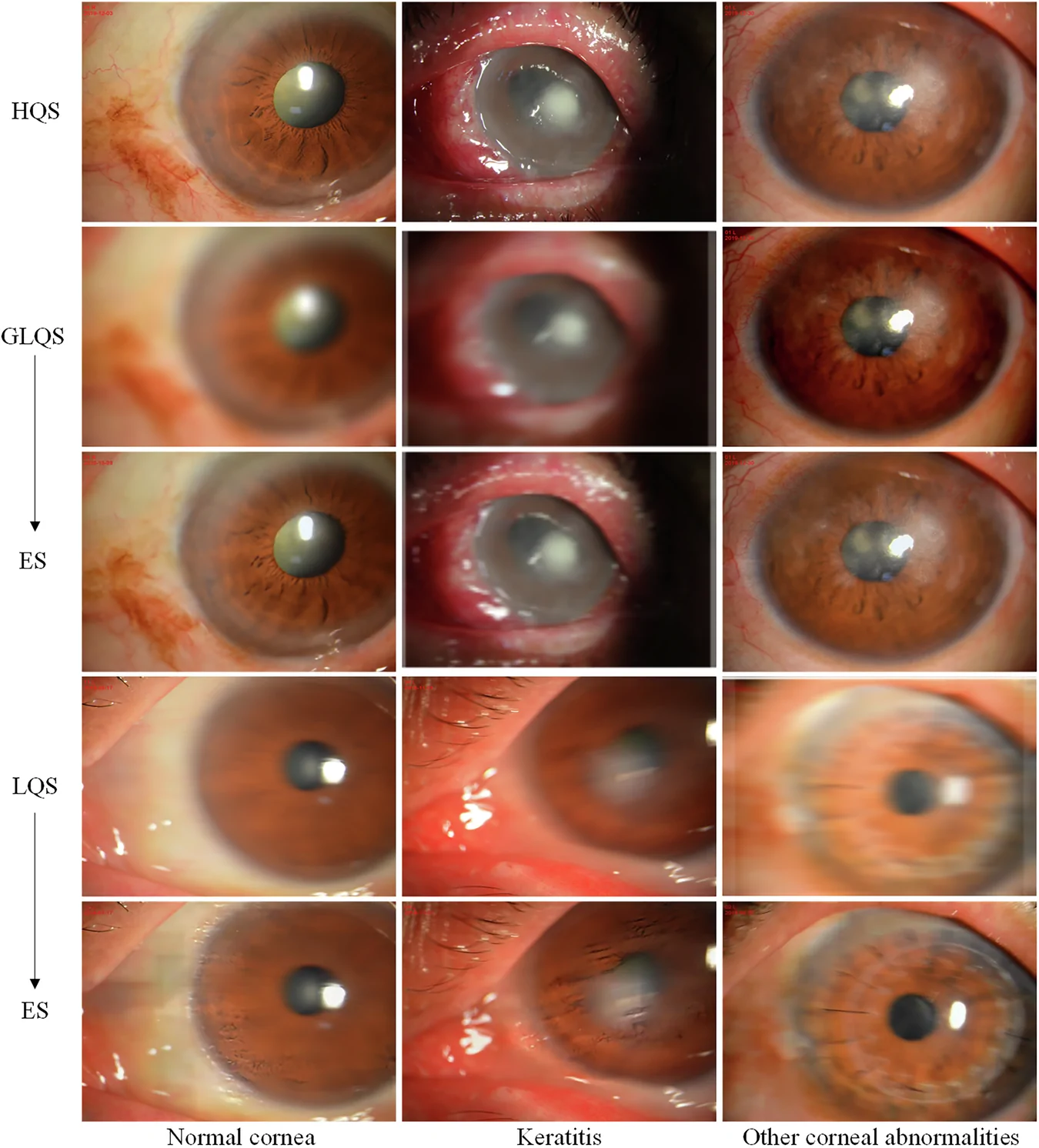
Qualitative comparison of representative LQS and ES images. HQS high-quality slit-lamp, LQS low-quality slit-lamp, GLQS generated low-quality slit-lamp, ES enhanced slit-lamp.

Figure 1 provides a detailed visual assessment of SCUNet’s enhancement capabilities on both GLQS and LQS images. The first three rows illustrate the enhancement effects on GLQS images: the first row shows the original HQS images, the second row displays the GLQS images generated by applying a degradation algorithm to the HQS images, and the third row presents the corresponding ES images restored by SCUNet. Notably, in the first (DB category) and third (ED category) columns, the ES images closely resemble the original HQS images. Additionally, in the second column (MB category), the ES image exhibits a smoother appearance, effectively alleviating the negative impact of the MB factor. The fourth and fifth rows focus on clinical LQS images. The fourth row presents real-world LQS images, while the fifth row displays the enhancement outcomes after applying the SCUNet model. In these clinical cases, the ES images reveal substantial improvements in clarity, particularly in critical regions such as ocular structures, lesions, and areas affected by complications. These qualitative enhancements align with the previously quantitative evaluation results, further confirming the effectiveness of the SCUNet in enhancing the quality of LQS images.

### Ablation experiments of AKDF-LQSI on the internal real-world LQS dataset

To systematically evaluate the contribution of key components within the AKDF-LQSI framework, we conducted a controlled ablation study focusing on two architectural innovations: GCT and SCUNet modules. This analysis was performed on the NEH real-world LQS test set under three distinct experimental configurations: (1) AKDF-GCT—a baseline implementation utilizing a GCT-enhanced DenseNet121 for direct classification of raw LQS images, excluding SCUNet preprocessing; (2) AKDF-SCUNet‌—a variant employing SCUNet-processed ES images as input to DenseNet121 without GCT integration; (3) AKDF-LQSI‌‌—the comprehensive framework incorporating SCUNet-based preprocessing alongside GCT-optimized classification.

As quantified in Table 2, the AKDF-LQSI model demonstrated superior diagnostic performance, achieving 91.8% accuracy (95% CI: 88.3–95.2), 92.8% specificity (95% CI: 88.7–96.9), and 90.0% sensitivity (95% CI: 83.8–96.2) for keratitis detection. This represents statistically significant improvements over both ablated configurations—4.6% absolute accuracy gain versus both AKDF-GCT and AKDF-SCUNet. Similar performance differentials were observed across normal cornea and other cornea abnormalities categories, confirming the framework’s robustness.

**Table 2:** Ablation experiment results of AKDF-LQSI using the internal NEH LQS test set

One-vs.-rest classification	NEH LQS test set			
	Accuracy (95% CI)		Sensitivity (95% CI)	Specificity (95% CI)
**Normal vs. keratitis+other**				
AKDF-GCT		88.5% (84.5–92.5)	73.2% (62.9–83.5)	94.8% (91.4–98.1)
AKDF-SCUNet		89.7% (85.9–93.5)	80.3% (71.0–89.5)	93.6% (89.9–97.3)
AKDF-LQSI		**89.7% (85.9–93.5)**	**84.5% (76.1–92.9)**	**91.9% (87.8–95.9)**
**Keratitis vs. other** **+** **normal**				
AKDF-GCT		87.2% (83.0–91.4)	88.9% (82.4–95.4)	86.3% (80.8–91.7)
AKDF-SCUNet		87.2% (83.0–91.4)	85.6% (78.3–92.8)	88.2% (83.1–93.3)
AKDF-LQSI		**91.8% (88.3–95.2)**	**90.0% (83.8–96.2)**	**92.8% (88.7–96.9)**
**Other vs. keratitis** **+** **normal**				
AKDF-GCT		84.0% (79.3–88.6)	75.6% (66.3–84.9)	88.2% (83.2–93.2)
AKDF-SCUNet		84.4% (79.8–88.9)	75.6% (66.3–84.9)	88.8% (84.0–93.7)
AKDF-LQSI		**95.5% (92.9–98.1)**	**90.2% (83.8–96.7)**	**98.1% (96.0–100)**

*NEH* Ningbo Eye Hospital, *LQS* low-quality slit-lamp, *AKDFLQSI* automated keratitis diagnosis framework for LQS images, *GCT* Gaussian context transformer, *SCUNet* U-Net with Swin-Conv module, *CI* confidence intervals.

**Fig. 2:**
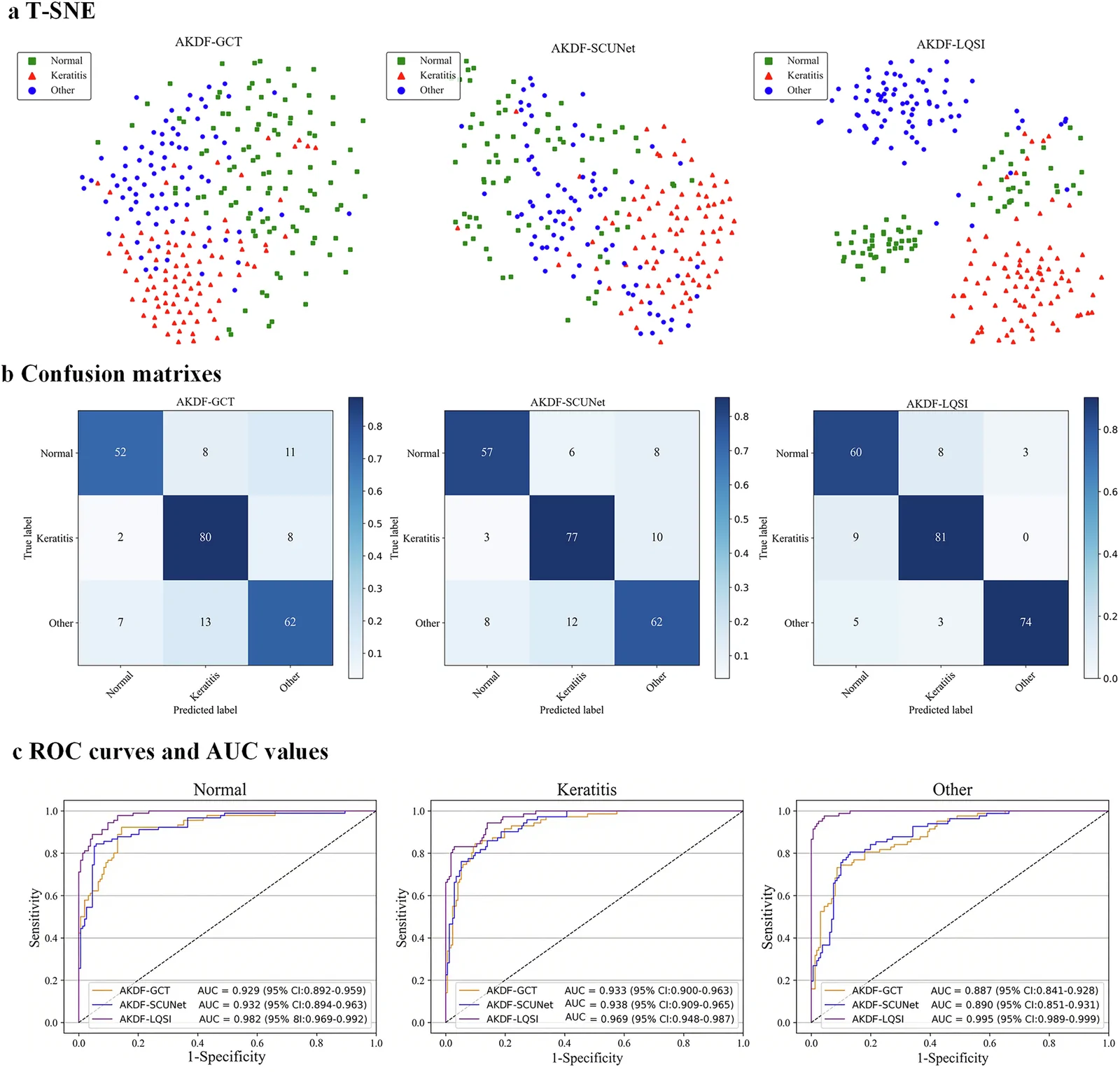
Performance comparison of AKDF-LQSI and baseline architectures for keratitis diagnosis on the internal NEH LQS test set. **a** Two-dimensional t-SNE visualization of embedding features learned by AKDF-LQSI and baseline architectures. Each point represents an individual sample, and distinct colored clusters correspond to different classes, illustrating feature separability. **b** Confusion matrices depicting the classification accuracies of AKDF-LQSI and baseline architectures. **c** ROC curves of AKDF-LQSI and baseline architectures for the identification of normal cornea, keratitis, and other cornea abnormalities. T-SNE t-distributed stochastic neighbor embedding, NEH Ningbo Eye Hospital, LQS low-quality slit-lamp, AKDF-LQSI automated keratitis diagnosis framework for LQS images, GCT Gaussian context transformer, SCUNet U-net with Swin-Conv module, *ROC* receiver operating characteristic, *AUC* area under the ROC curve.

T-SNE visualization^42^ of penultimate layer activations (Fig. 2a) revealed critical architectural impacts. While AKDF-GCT and AKDF-SCUNet exhibited inter-class overlap, AKDF-LQSI achieved near-optimal class separation, demonstrating tight per-class clustering. This geometric alignment with diagnostic categories substantiates the synergistic interaction between SCUNet’s enhancement capabilities and GCT’s feature refinement.

Confusion matrix analysis (Fig. 2b) characterized misclassification distributions across diagnostic categories. AKDF-GCT reduced the false negatives‌ of keratitis by 23.1% compared to AKDF-SCUNet (13 vs. 10 instances) at the cost of increased normal cornea errors (14 to 19). The AKDF-LQSI model optimized this tradeoff, limiting misclassifications to 11 (normal), 9 (keratitis), and 8 (other cornea abnormalities) cases while preserving inter-class diagnostic equilibrium. Quantitative ROC curves (Fig. 2c) and PR curves (Supplementary Fig. 1a) further confirms AKDF-LQSI’s diagnostic superiority, coupled with a near-perfect AUC of 0.969‌ [95% CI: 0.948–0.987] for keratitis and 0.995‌ [95% CI: 0.989–0.999] for other cornea abnormalities. This evidences rigorously optimized sensitivity-specificity equilibrium across all three classification categories.

### Ablation experiments of AKDF-LQSI on the external real-world LQS dataset

To assess the generalizability of AKDF-LQSI, we conducted ablation experiments on the external real-world LQS dataset representing diverse imaging protocols and demographic variability. As detailed in Table 3, AKDF-LQSI maintained robust diagnostic performance despite domain shifts, achieving significant improvements over baseline models. For keratitis, AKDF-LQSI achieved an accuracy of ‌92.5% (95% CI: 90.1–94.9), sensitivity‌ of ‌85.7% (79.8–91.7), and ‌ specificity‌ of 95.3% (93.0–97.6), respectively, surpassing suboptimal AKDF-GCT by 5.3, 9.0, and 3.7%. Consistent performance improvements were also observed in the categories of normal cornea and other cornea abnormalities. These results confirmed that AKDF-LQSI demonstrated robust performance even on a real-world LQS dataset.

**Table 3:** Ablation experiment of AKDF-LQSI using the external LQS test set

One-vs.-rest classification	External LQS test set			
	Accuracy (95% CI)		Sensitivity (95% CI)	Specificity (95% CI)
**Normal vs. keratitis** **+** **other**				
AKDF-GCT		79.5% (75.8–83.2)	66.7% (59.2–74.1)	86.0% (82.1–90.0)
AKDF-SCUNet		77.4% (73.5–81.2)	64.7% (57.1–72.3)	83.8% (79.6–87.9)
AKDF-LQSI		**89.6% (86.8–92.4)**	**76.5% (69.7–83.2)**	**96.3% (94.2–98.5)**
**Keratitis vs. other** **+** **normal**				
AKDF-GCT		87.2% (84.2–90.3)	76.7% (69.5–83.9)	91.6% (88.6–94.6)
AKDF-SCUNet		86.4% (83.2–89.5)	74.6% (67.3–82.0)	91.3% (88.2–94.4)
AKDF-LQSI		**92.5% (90.1–94.9)**	**85.7% (79.8–91.7)**	**95.3% (93.0–97.6)**
**Other vs. keratitis** **+** **normal**				
AKDF-GCT		73.3% (69.3–77.4)	67.9% (60.8–74.9)	76.6% (71.7–81.5)
AKDF-SCUNet		74.7% (70.7–78.7)	69.0% (62.1–76.0)	78.0% (73.3–82.8)
AKDF-LQSI		**85.7% (82.5–88.9)**	**89.3% (84.6–94.0)**	**83.6% (79.3–87.9)**

*LQS* low-quality slit-lamp, *AKDF-LQSI* automated keratitis diagnosis framework for LQS images, *GCT* Gaussian context transformer, *SCUNet* U-net with Swin-Conv module, *CI* confidence intervals.

**Fig. 3:**
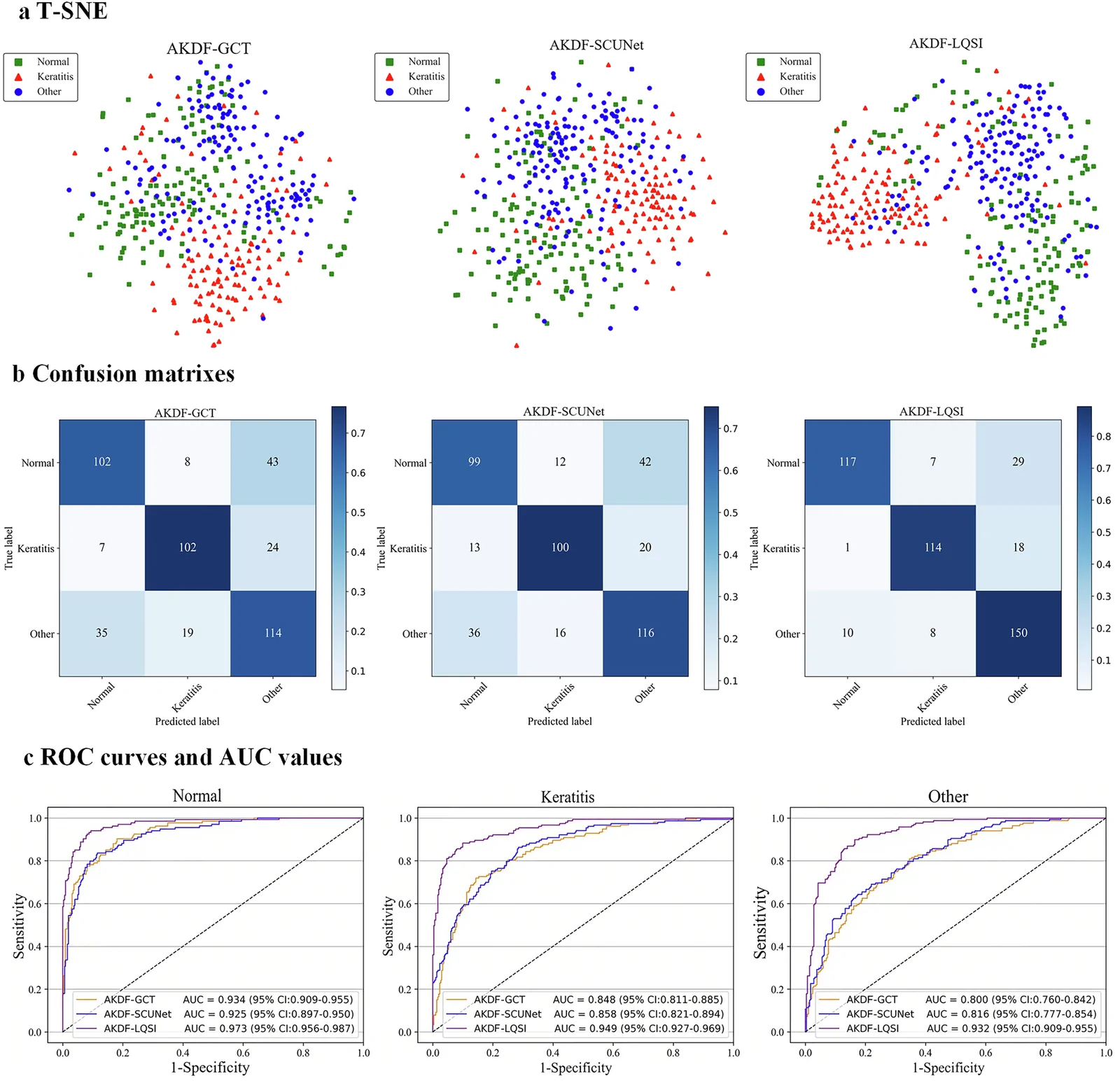
Performance comparison of AKDF-LQSI and baseline architectures for keratitis diagnosis on the external LQS test set. **a** Two-dimensional t-SNE visualization of embedding features learned by AKDF-LQSI and baseline architectures. Each point represents an individual sample, and distinct colored clusters correspond to different classes, illustrating feature separability. **b** Confusion matrices depicting the classification accuracies of AKDF-LQSI and baseline architectures. **c** ROC curves of AKDF-LQSI and baseline architectures for the identification of normal cornea, keratitis, and other cornea abnormalities. T-SNE t-distributed stochastic neighbor embedding, LQS low-quality slit-lamp, AKDF-LQSI automated keratitis diagnosis framework for LQS images, GCT Gaussian context transformer, SCUNet U-net with Swin-Conv module, ROC receiver operating characteristic, AUC area under the ROC curve.

Complementary feature-space analysis (Fig. 3a) revealed AKDF-LQSI’s superior discriminative capability, characterized by tighter intra-class clustering‌ and reduced inter-class overlap‌. This geometric superiority translated into practical diagnostic improvements, with AKDF-LQSI reducing overall misclassification rates by ‌46.3%‌ (73 vs. 136 for AKDF-GCT), particularly enhancing keratitis detection (‌19 false negatives‌ vs. 31 for AKDF-GCT). ‌ Confusion matrices (Fig. 3b), ROC curves (Fig. 3c), and PR curves (Supplementary Fig. 1b) further confirmed AKDF-LQSI’s cross-domain reliability, achieving an AUC of ‌0.949 (95% CI: 0.927–0.969) for keratitis detection—an absolute improvement of 0.101 over AKDF-GCT. These findings substantiate the dataset-agnostic generalization of AKDF-LQSI, highlighting its potential for real-world clinical deployment.

### Interpretability analysis of AKDF-LQSI

Exploring model interpretability is essential for clinical translation and has been the focus of extensive researches^43–46^. To investigate the decision-making mechanisms of AKDF-LQSI in keratitis diagnostics, we integrated Grad-CAM (gradient-weighted class activation mapping)^47^ and SHAP (SHapley Additive exPlanations)^48^ to visualize the contribution of image regions to model predictions. Grad-CAM generates spatial attention maps by backpropagating the gradient of the predicted class through the convolutional feature maps, highlighting regions with the greatest influence on the output. SHAP, grounded in cooperative game theory, estimates the marginal contribution of each input feature (pixel or region) to the predicted class, producing maps where positive values indicate supportive regions and negative values indicate opposing influence.

**Fig. 4:**
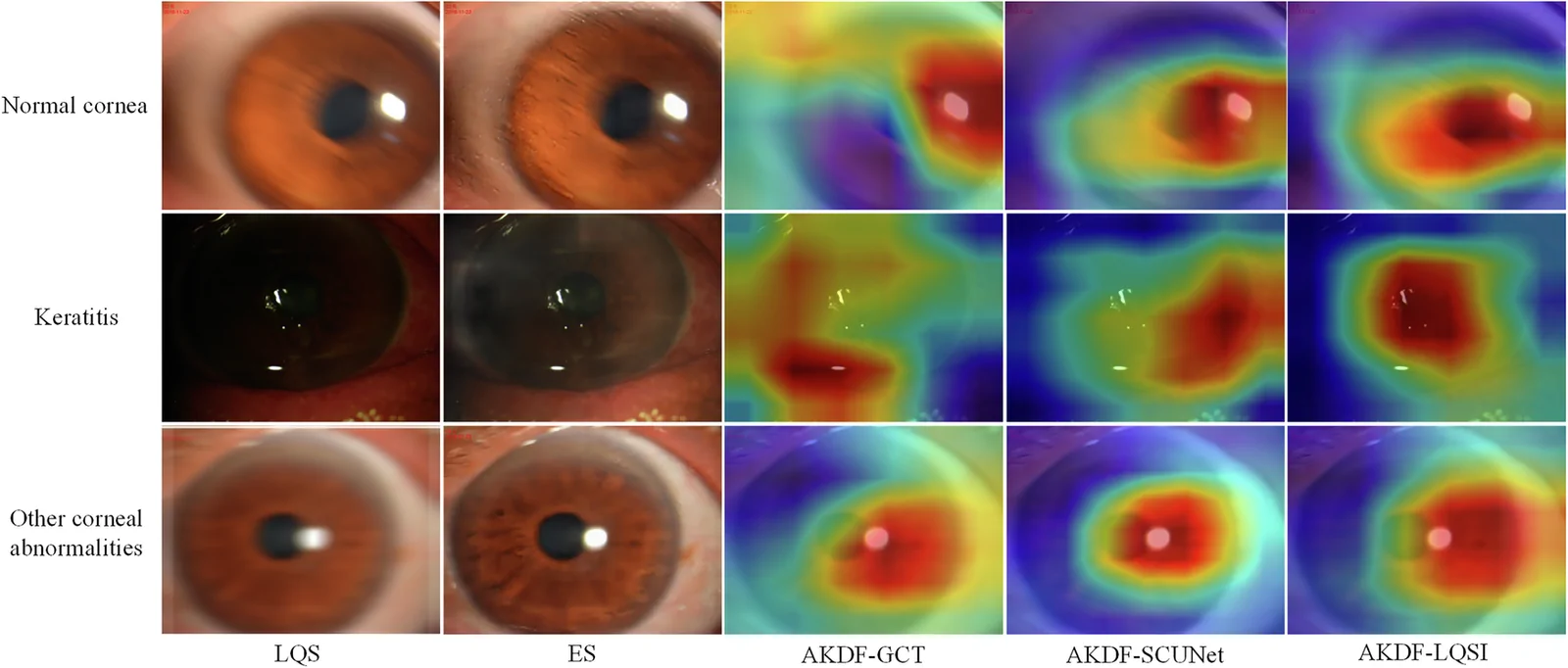
Interpretability comparison of heatmaps for AKDF-GCT, AKDF-SCUNet, and AKDF-LQSI. LQS low-quality slit-lamp, ES enhanced slit-lamp, AKDF-LQSI automated keratitis diagnosis framework for LQS images. GCT Gaussian context transformer, SCUNet U-net with Swin-Conv module.

As shown in Fig. 4 and Supplementary Fig. 2, for correctly classified samples, AKDF-LQSI consistently identified clinically relevant structures. In a motion-blurred normal cornea, Grad-CAM primarily highlighted the corneal region, whereas SHAP additionally emphasized adjacent conjunctival regions, reflecting a broader anatomical context. In the exposure-distorted keratitis case, both methods accurately identified corneal lesions and surrounding conjunctival complications. For other cornea abnormalities, Grad-CAM and SHAP focused on lesions and nearby pathological regions, effectively capturing key diagnostic areas.

Furthermore, our analysis provided insight into patterns underlying misclassification (Supplementary Figs. 3 and 4). For the keratitis sample misclassified as other corneal abnormalities, Grad-CAM exhibited dispersed attention across conjunctival regions, while SHAP highlighted peripheral conjunctiva contributions, suggesting that critical lesions were underrepresented in the model’s decision. Conversely, other cornea abnormalities misclassified as normal displayed Grad-CAM focus on predominantly healthy tissue with partial lesions, whereas SHAP attributed positive contributions to normal cornea structures, revealing the sources of misclassification.

By combining Grad-CAM and SHAP, our approach captures both the spatial focus and the quantitative contribution of each region to predictions. These complementary insights elucidate how AKDF-LQSI leverages enhanced structural information from ES images to distinguish keratitis and other cornea conditions, while also revealing sources of potential misclassification, thereby providing mechanistic interpretability to support clinical deployment.

### Comparison and statistical analysis of AKDF-LQSI vs. ophthalmologists

**Fig. 5:**
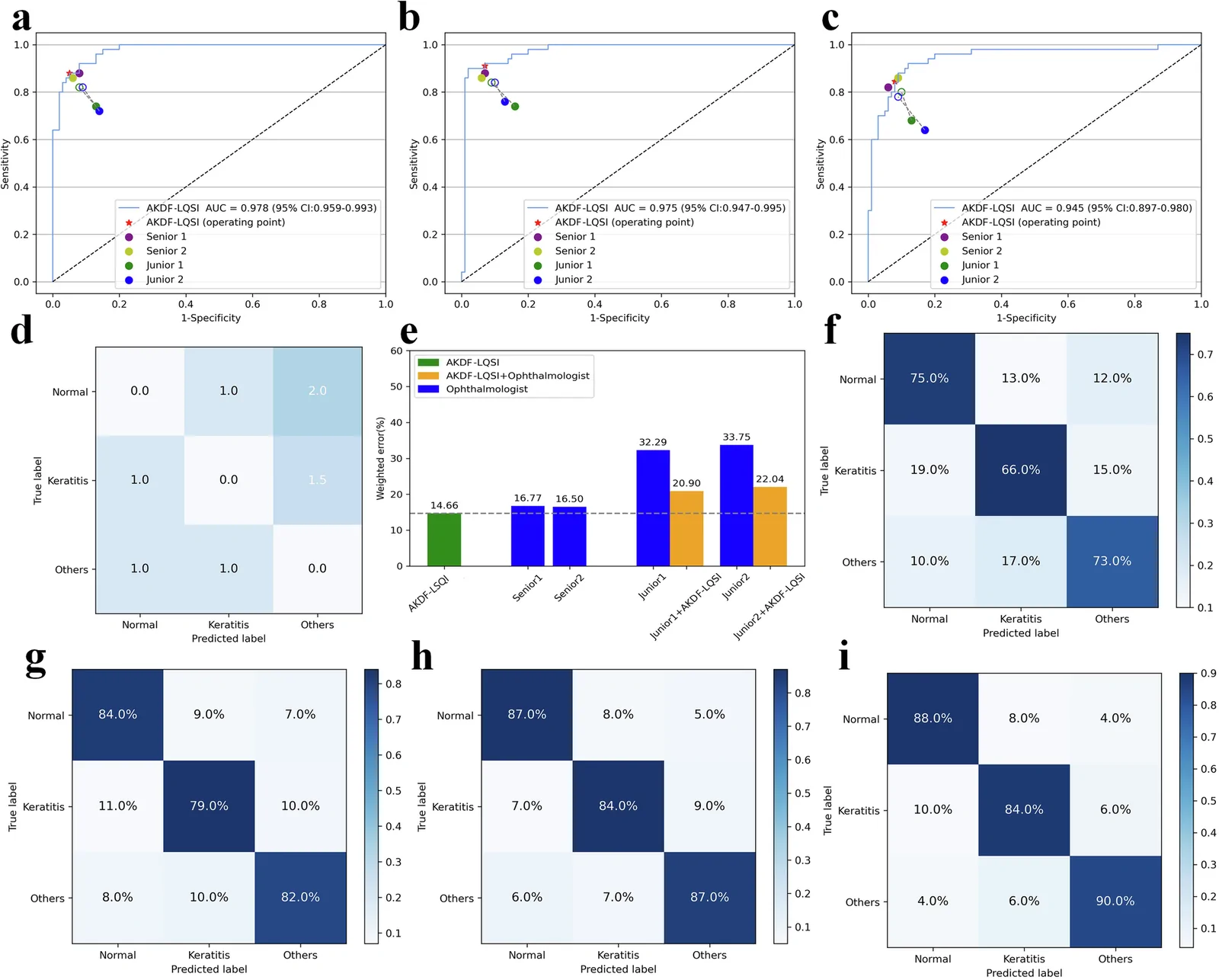
Performance comparison of AKDF-LQSI and ophthalmologists. **a**–**c** ROC curves illustrating the diagnostic performance of AKDF-LQSI and four ophthalmologists (two junior and two senior) for detecting normal cornea (**a**), keratitis (**b**), and other cornea abnormalities (**c**). Solid markers denote the unassisted performance of junior and senior ophthalmologists, whereas hollow markers indicate the performance of junior ophthalmologists with AKDF-LQSI support. **d** Penalty score matrix is applied to weight different misclassification classes. **e** Weighted error rates calculated based on the penalty score matrix. **f** Average confusion matrix of two junior ophthalmologists. **g** Average confusion matrix of two junior ophthalmologists with AKDF-LQSI assistance. **h** Average confusion matrix of two senior ophthalmologists. **i** Confusion matrix of AKDF-LQSI. AKDF-LQSI automated keratitis diagnosis framework for low-quality slit-lamp images, AUC area under the receiver operating characteristic curve, CI confidence intervals.

A blinded diagnostic trial was conducted using 150 slit-lamp images (50 normal cornea, 50 keratitis, and 50 other cornea abnormalities) randomly selected from the NEH LQS test set. Four board-certified ophthalmologists were stratified into junior (6–15 years of clinical experience) and senior (16–25 years) cohorts for comparative analysis with AKDF-LQSI. Diagnostic performance was quantified using ROC curves (Fig. 5a–c), average confusion matrices (Fig. 5f–i), and weighted error rates (WER, Fig. 5e) derived from a clinical severity-based penalty matrix (Fig. 5d).

The AKDF-LQSI demonstrated superior diagnostic accuracy compared to junior ophthalmologists and achieved performance parity with senior ophthalmologists (Fig. 5a–c and Table 4). To quantify misclassification impact, a penalty matrix (Fig. 5d) was applied, assigning differential weights to errors based on clinical severity. The AKDF-LQSI exhibited a WER of 14.66%, significantly lower than the ophthalmologists’ average WER of 24.83% (range: 16.50–33.75%, Fig. 5e).

**Table 4:** Comparison of AKDF-LQSI with ophthalmologists in the classification of normal cornea, keratitis, and other cornea abnormalities

One-vs.-rest classification	AKDF-LQSI	Senior1	Senior2	Junior1	Junior2	Junior1 + AKDF-LQSI	Junior2 + AKDF-LQSI	P1	P2	P3	P4	P5	P6
**Normal vs. keratitis** **+** **othe**r													
Accuracy (95% CI)	93.3%(89.3–97.3)	90.7% (86.0–95.3)	91.3% (86.8–95.8)	82.7% (76.6–88.7)	81.3% (75.1–87.6)	88.7% (83.6–93.7)	88.0% (82.8–93.2)	0.002	0.008	1.000	1.000	0.009	0.002
Sensitivity (95% CI)	90.0% (81.7–98.3)	88.0% (79.0–97.0)	86.0% (76.4–95.6)	74.0% (61.8–86.2)	72.0% (59.6–84.4)	82.0% (71.4–92.6)	82.0% (71.4–92.6)	0.092	0.031	1.000	1.000	0.118	0.146
Specificity (95% CI)	95.0% (90.7–99.3)	92.0% (86.7–97.3)	94.0% (89.3–98.7)	87.0% (80.4–93.6)	86.0% (79.2–92.8)	92.0% (86.7–97.3)	91.0% (85.4–96.6)	0.012	0.146	1.000	1.000	0.064	0.006
**Keratitis vs. other** **+** **normal**													
Accuracy (95% CI)	91.3% (86.8–95.8)	91.3% (86.8–95.8)	91.3% (86.8–95.8)	80.7% (74.3–87.0)	83.3% (77.4–89.3)	88.7% (83.6–93.7)	88.0% (82.8–93.2)	<0.001	<0.001	0.424	0.375	0.008	0.004
Sensitivity (95% CI)	88.0% (79.0–97.0)	88.0% (79.0–97.0)	86.0% (76.4–95.6)	74.0% (61.8–86.2)	76.0% (64.2–87.8)	84.0% (73.8–94.2)	84.0% (73.8–94.2)	0.008	0.022	1.000	0.625	0.039	0.070
Specificity (95% CI))	93.0% (88.0–98.0)	93.0% (88.0–98.0)	94.0% (89.3–98.7)	84.0% (76.8–91.2)	87.0% (80.4–93.6)	91.0% (85.4–96.6)	90.0% (84.1–95.9)	0.039	0.012	0.375	1.000	0.180	0.065
**Other vs. keratitis** **+** **normal**													
Accuracy (95% CI)	90.0% (85.2–74.8)	90.0% (85.2–94.8)	89.3% (84.4–94.3)	80.7% (74.3–87.0)	76.7% (69.9–83.4)	86.7% (81.2–92.1)	86.7% (81.2–92.1)	0.001	<0.001	1.000	1.000	0.013	0.011
Sensitivity (95% CI)	84.0% (73.8–94.2)	82.0% (71.4–92.6)	86.0% (76.4–95.6)	68.0% (55.1–80.9)	64.0% (50.7–77.3)	80.0% (68.9–91.1)	78.0% (66.5–89.5)	0.008	0.002	1.000	1.000	0.092	0.013
Specificity (95% CI)	93.0% (88.0–98.0)	94.0% (89.3–98.7)	91.0% (85.4–96.6)	87.0% (80.4–93.6)	83.0% (75.6–90.4)	90.0% (84.1–95.9)	91.0% (85.4–96.6)	0.109	0.006	1.000	0.688	0.118	0.388

Normal normal cornea, Other other cornea abnormalities, *CI* confidence interval, *P1* indicates the *p* value calculated between AKDF-LQSI and junior1 using the McNemar test. *P2* indicates the *p* value calculated between AKDF-LQSI and junior2 using the McNemar test. *P3* indicates the *p* value calculated between AKDF-LQSI and senior1 using the McNemar test. *P4* indicates the *p* value calculated between AKDF-LQSI and senior2 using the McNemar test. *P5* indicates the *p* value calculated between the junior1 and “junior1 + AKDF-LQSI” using the McNemar test, *P6* indicates the *p* value calculated between the junior2 and “junior2 + AKDF-LQSI” using the McNemar test.

Analysis of misclassified cases revealed complementary diagnostic patterns between AKDF-LQSI and clinicians. The AKDF-LQSI correctly diagnosed six cases misclassified by all four ophthalmologists (Supplementary Table 4 and Supplementary Fig. 5a), while conversely, five cases correctly identified by all clinicians were misclassified by AKDF-LQSI (Supplementary Table 4 and Supplementary Fig. 5b). Though no systematic error patterns emerged, the presence of borderline cases suggests potential complementary roles for human–AI collaboration in keratitis diagnosis.

To evaluate AKDF-LQSI’s assistive potential, a crossover study was conducted. Four weeks after initial assessments, junior ophthalmologists repeated diagnoses with access to the AKDF-LQSI’s classification probabilities (Representative examples in Supplementary Fig. 6). Junior ophthalmologists showed significant performance improvement when aided by AKDF-LQSI, with an average WER decreasing from 33.02 to 21.47% (Fig. 5e). Notably, the accuracy of junior ophthalmologists in detecting keratitis, normal cornea, and other cornea abnormalities improved significantly (*P* < 0.05, Table 4). Additionally, comparative confusion matrices (Fig. 5f–i) illustrate this enhancement, with assisted junior clinicians approaching senior clinician performance.

## Discussion

This study proposed AKDF-LQSI, a novel algorithm for the automatic diagnosis of keratitis from LQS images. By embedding the Swin-Conv module into a U-Net framework, we developed SCUNet, an innovative GAN model designed to improve the capability of mapping LQS images to HQS counterparts. Quantitative assessments and qualitative comparisons demonstrated the effectiveness of SCUNet in enhancing image quality. Furthermore, we proposed ‌GCT-DenseNet‌, a DenseNet variant augmented with the GCT module, which extracted discriminative features for keratitis classification. Ablation experiments confirmed the feasibility of SCUNet in improving the quality of LQS images, as well as the efficacy of the GCT module in boosting the classifier performance on ES images. The generalization of AKDF-LQSI was validated across internal and external datasets. Heatmap visualizations of the model’s decision-making process provided insights into the alignment of its predictions with relevant lesions and complications. Statistical comparisons between ophthalmologists and AI models revealed that the AKDF-LQSI outperformed junior ophthalmologists, achieving diagnostic performance comparable to that of senior ophthalmologists.

In recent years, numerous advanced studies have emerged focusing on the automatic screening and grading of keratitis using slit-lamp images^7–9,31,35–40^. Experimental results have demonstrated that existing algorithms, when applied to HQS images, achieved performance comparable to that of senior ophthalmologists. However, these studies predominantly relied on pre-selected HQS images, neglecting challenges posed by LQS images. LQS images are inevitable in clinical settings, presenting significant barriers to the widespread clinical adoption of these models. To address this limitation, this study proposed a novel SCUNet model to effectively enhance the quality of LQS images. Furthermore, the development of the GCT-DenseNet was applied to improve the automatic diagnosis of keratitis based on ES images. This approach offers a promising solution for addressing the challenges posed by LQS images in real-world clinical applications.

To evaluate the clinical performance of the AKDF-LQSI, this study compared its diagnostic capabilities with those of ophthalmologists of varying expertise levels. The experimental results indicated that the AKDF-LQSI performed comparably to senior ophthalmologists and significantly improved the diagnostic accuracy of junior ophthalmologists (Fig. 5). These findings suggest that, in real-world clinical settings, AKDF-LQSI can reduce dependence on HQS imaging data acquisition, thereby alleviating the need for specialized expertise in resource-limited or remote areas. This highlights the potential of the AKDF-LQSI to promote equitable distribution of healthcare access and enhance care quality in underserved regions.

Compared to previous studies, this study introduces several critical advancements. First, the AKDF-LQSI model is specifically designed to address the challenges posed by LQS images, whereas most existing researches rely on HQS images. By leveraging the SCUNet model, this study effectively addresses clinical image degradation, such as MB, DB, and ED. The SCUNet model enables ES images to exhibit clarity and detail comparable to HQS images, thereby providing more reliable inputs for classifiers. Second, the integration of the GCT module significantly enhances the ability of GCT-DenseNet121 to capture global contextual information. The GCT module strengthens the representation of clinically relevant lesions and complications within slit-lamp images while reducing noise interference. Third, extensive ablation experiments reveal the crucial role of both the SCUNet and GCT modules in enhancing the performance of the automated diagnostic model. Results from external dataset experiments confirm the generalizability of the AKDF-LQSI model. Fourth, the AKDF-LQSI exhibits clinically feasible computational efficiency, requiring less than 100 ms per image to perform both enhancement and classification despite its substantial training time (Supplementary Table 2), highlighting its potential for real-time clinical deployment. Finally, the human-machine comparison experiment reveals that the AKDF-LQSI matches the diagnostic capabilities of senior ophthalmologists, while also aiding junior ophthalmologists in improving their diagnostic accuracy.

This study has several limitations. First, the AKDF-LQSI was primarily designed and validated for three common types of degradation in LQS images: MB, DB, and ED, and its applicability to less common degradation types remain to be explored. Second, the current work focuses on the automatic diagnosis of normal cornea, keratitis, and other corneal abnormalities. As more LQS images of keratitis subtypes are annotated, future work will explore the applicability of the proposed framework for fine-grained keratitis subtyping, incorporating weighted loss functions and targeted data augmentation to address potential class imbalance and long-tail distributions. In addition, further efforts are needed to explore lightweight models, integrate multimodal clinical data, and conduct multicenter prospective studies with federated learning to ensure real-time performance, generalizability, and privacy-preserving clinical deployment. Despite these limitations, the proposed AKDF-LQSI offers a practical and feasible strategy for the automatic diagnosis of keratitis from LQS images, demonstrating promising generalization performance across multicenter datasets.

## Methods

### Dataset

The dataset utilized for the development of the AKDF-LQSI model was derived from routine examinations conducted at NEH, affiliated with Wenzhou Medical University, between January 2017 and June 2024. The NEH dataset consists of 6567 HQS images from 3568 individuals and 1619 LQS images from 1002 individuals. These images were independently and randomly partitioned into training, validation, and test sets in a 70:15:15% ratio to facilitate model training and evaluation. Images from the same individual were assigned to only one same set to prevent data leakage and ensure unbiased performance evaluation. To further assess model generalizability, an external dataset was collected from ZEH, JEH, and NOC under consistent inclusion criteria, comprising a total of 1858 HQS images from 1182 individuals and 454 LQS images from 326 individuals. Detailed dataset characteristics are provided in Table 5. Notably, GLQS images were generated by applying degradation algorithms to each original HQS image.

**Table 5:** Distributions of HQS and LQS images from NEH and external clinical institutions

Item	NEH HQS dataset			NEH LQS dataset			External HQS dataset	External LQS dataset
Total no. of images	6567			1619			1858	454
No. of subjects	3568			1002			1182	326
Age, mean/range (years)	41.6/4–98			42.4/4–92			42.8/5-96	44.3/5–92
No. (%) of women	1689 (47.3)			483 (48.2)			581 (49.2)	162 (49.7)
Camera model	Canton Optics LS-7						Sanyo VPC-MZ3G, Kanghua SLM-3, Nikon DSC D5200	
	Training set	Validation set	Test set	Training set	Validation set	Test set	Test set	Test set
Normal cornea	1756	408	373	330	71	71	628	153
Keratitis	2185	511	483	421	91	90	756	133
Other cornea abnormalities	585	136	130	381	82	82	474	168
GLQS images	4526	1055	986	-	-	-	1858	-

The NEH HQS and GLOS datasets are used to train and internally evaluate the SCUNet model, whereas external HQS and GLQS datasets support its generalization assessment. The SCUNet-enhanced NEH LQS dataset is then used to train and internally evaluate GCT-DenseNet121, with the external LQS dataset providing independent generalization testing.

*HQS* high-quality slit-lamp, *LQS* low-quality slit-lamp, *GLQS* generated low-quality slit-lamp, *NEH* Ningbo Eye Hospital.

### Image categories and reference standards

HQS images were annotated after filtering out blurry images. Using this curated HQS dataset, automatic keratitis diagnosis systems were developed, as detailed in our previous studies^7–9,30^. LQS images were characterized by various types of blurs, including MB, DB, and ED. Both HQS and LQS images were classified into three categories—normal cornea, keratitis, and other cornea abnormalities—based on comprehensive medical records, including clinical manifestations, corneal examinations, and follow-up assessments. Other cornea abnormalities include corneal dystrophies, corneal degeneration, corneal tumors, pterygium, and other non-keratitis corneal disorders. Images with uncertain diagnoses were excluded from the study.

This study involved two ophthalmologists with over 5 years of clinical experience to perform dual-blind annotations. The label for each image was assigned through consensus between the two ophthalmologists. In cases of disagreement, a senior cornea specialist with more than twenty years of clinical experience made the final determination. This rigorous annotation process ensured the accuracy and reliability of the image labels.

**Fig. 6:**
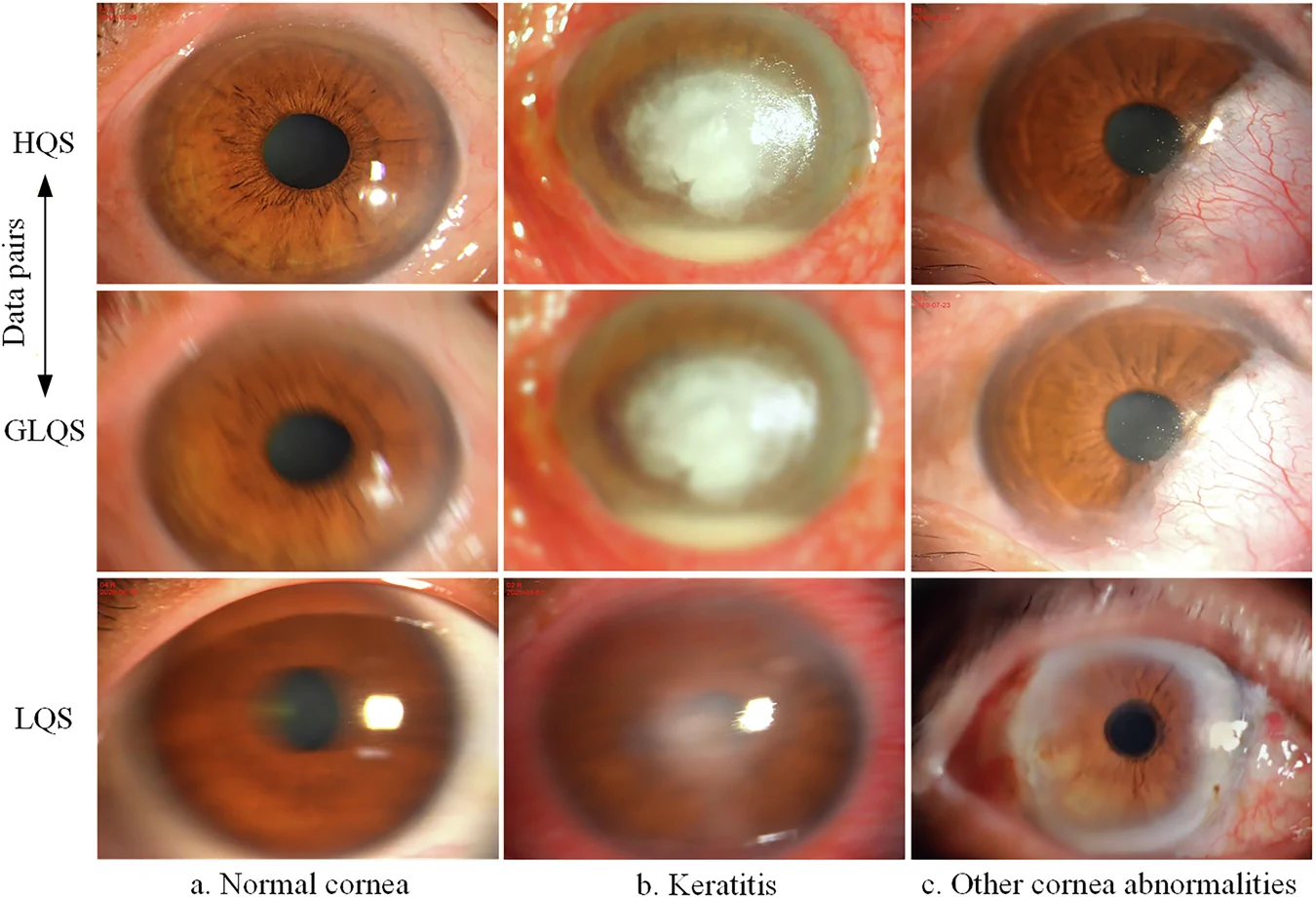
Representative HQS, GLQS, and LQS images of normal cornea, keratitis, and other cornea abnormalities. HQS high-quality slit-lamp, GLQS generated low-quality slit-lamp, LQS low-quality slit-lamp.

Figure 6 presents representative HQS and LQS images for keratitis. From left to right, the three columns represent the normal cornea, keratitis, and other cornea abnormalities, respectively. From top to bottom, the first row shows HQS images captured in real clinical settings. The second row displays three types of GLQS images generated by randomly applying a degradation algorithm to the first-row images. Specifically, motion blur manifests as linear or arc-shaped distortion; defocus blur typically results in overall fuzziness, characterized by the absence of sharp edges and fine details; exposure distortion is marked by uneven luminance distribution. The third row illustrates real-world LQS images.

To ensure the consistency between the GLQS and real LQS images, we performed pixel- and feature-level comparisons using histogram analysis and t-SNE visualization (Supplementary Fig. 7). Pixel-intensity histograms across keratitis, normal cornea, and other cornea abnormalities exhibited substantial overlap between generated and real LQS images, indicating similar intensity attenuation and contrast loss. At the feature level, t-SNE visualization of DenseNet121 embeddings from randomly sampled 400 generated images and 400 real LQS images per class revealed closely intermixed clusters, with no discernible separation in high-dimensional space. These quantitative comparisons confirm that the proposed degradation algorithm reliably reproduces real-world LQS artifacts across clinical centers.

### Overall framework of AKDF-LQSI

**Fig. 7:**
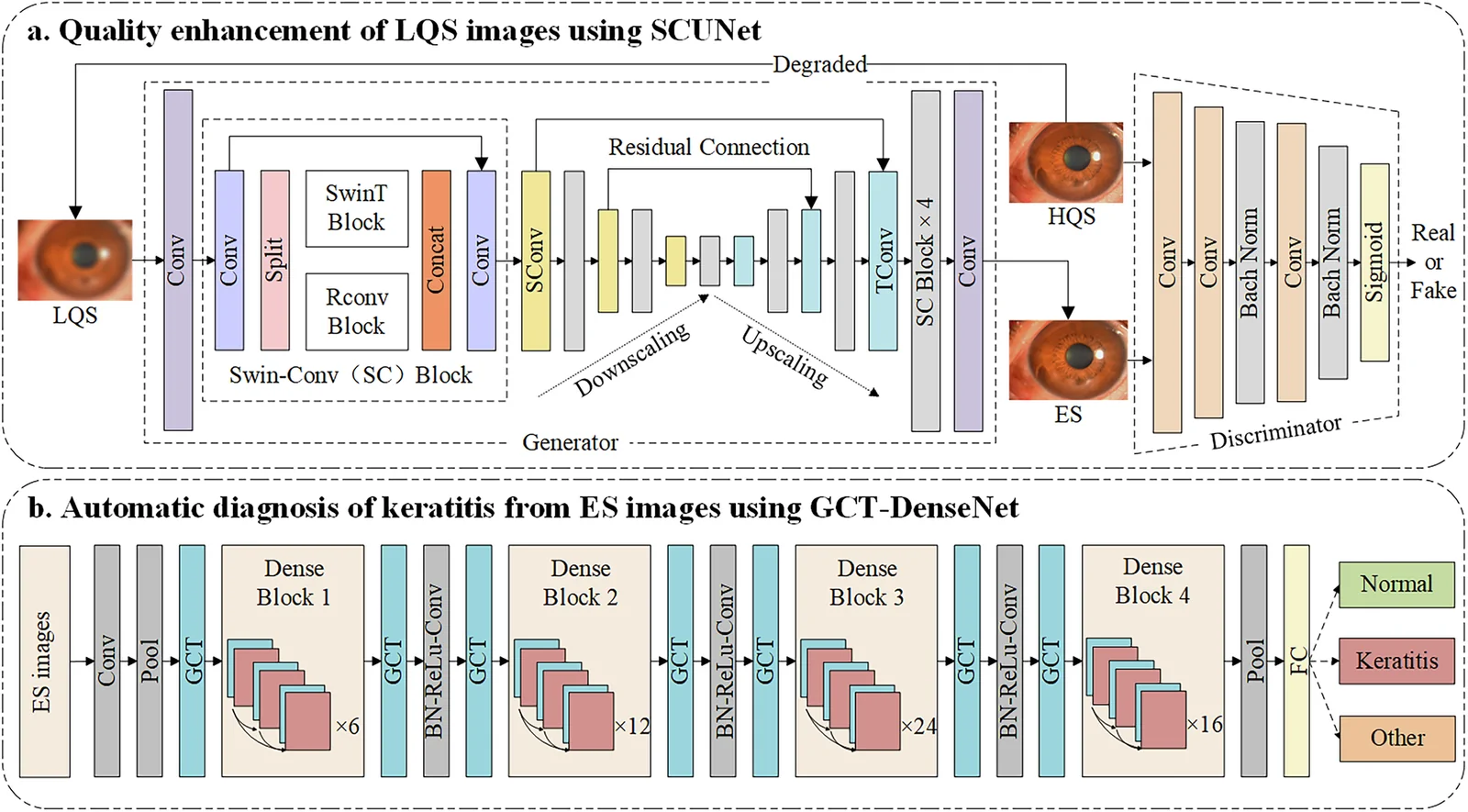
Overall framework of AKDF-LQSI. **a** Enhancement of LQS images using SCUNet to generate high-fidelity ES images. **b** Automatic diagnosis of keratitis from ES images using GCT-DenseNet. HQS high-quality slit-lamp, LQS low-quality slit-lamp, ES enhanced slit-lamp, GCT Gaussian context transformer, SCUNet U-Net with Swin-Conv module.

The AKDF-LQSI involves two key stages: quality enhancement of LQS images using SCUNet (Fig. 7a), and automatic diagnosis of keratitis through an improved convolutional neural network (CNN) applied to the ES images (Fig. 7b). LQS images are often plagued by various types of blur noise, which inevitably obscure keratitis lesions and associated complications. Thus, image quality enhancement is essential as a preprocessing step. In the first stage, HQS images are randomly degraded using a degradation algorithm to generate GLQS images, constructing the HQS and GLQS image pairs required for the GAN model. A novel GAN model, SCUNet, is then proposed by embedding the Swin-Conv (SC)^49^ module into the U-Net^50^ architecture to enhance LQS images. In the second stage, the ES images are fed into the GCT-DenseNet, an improved DenseNet121 network integrated with a Gaussian context transformer^51^ module, to identify normal cornea, keratitis, and other cornea abnormalities.

**Fig. 8:**
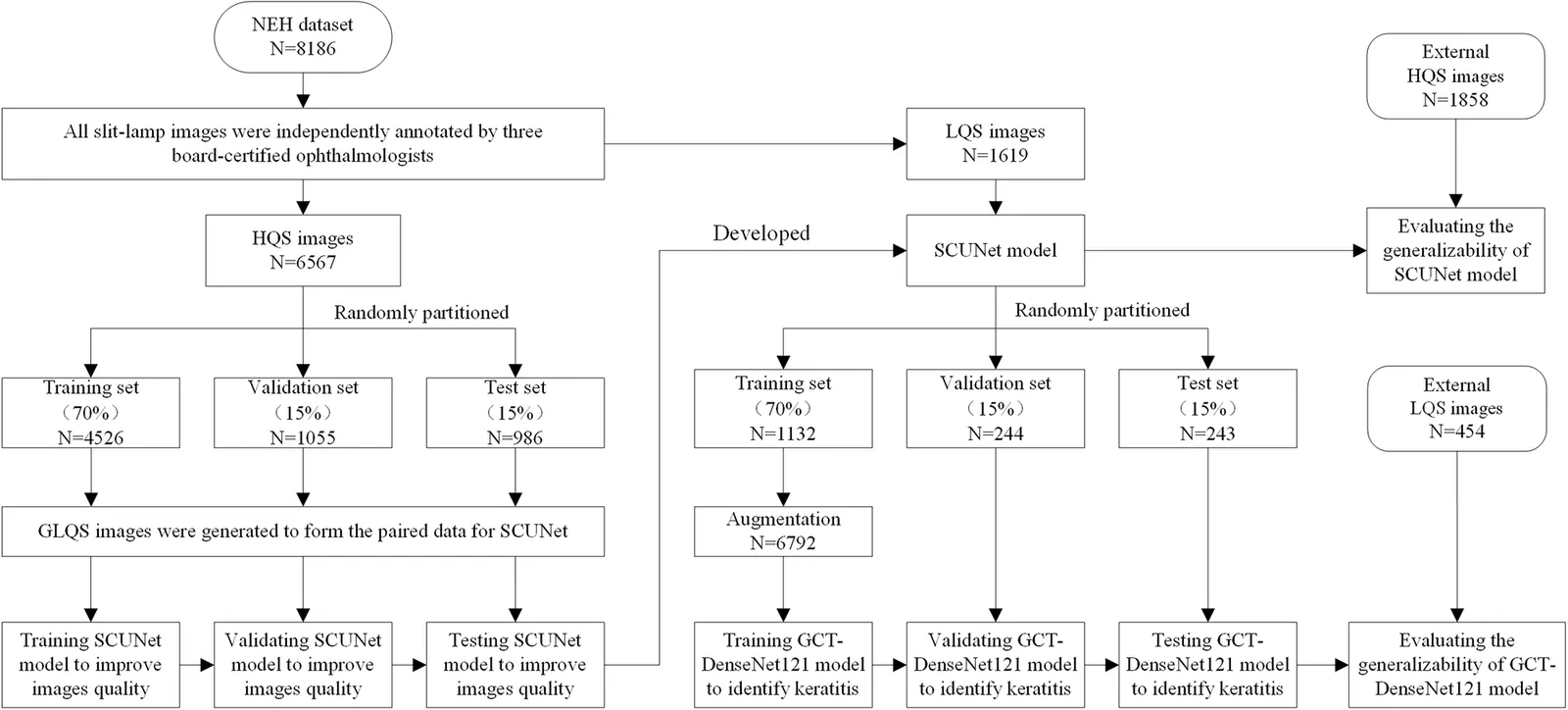
Development and evaluation flowchart of AKDF-LQSI. NEH Ningbo Eye Hospital, HQS high-quality slit-lamp. LQS low-quality slit-lamp, GLQS generated low-quality slit-lamp, GCT Gaussian context transformer, SCUNet U-net with Swin-Conv module.

The detailed development and evaluation flowchart of AKDF-LQSI is shown in Fig. 8. Initially, 8186 slit-lamp images from NEH were annotated by ophthalmologists as HQS images (6567) and LQS images (1619), and categorized by disease types (normal cornea, keratitis, and other cornea abnormalities). HQS images were degraded using a degradation algorithm to generate corresponding image pairs for training the SCUNet model. After training the SCUNet model, LQS test images from NEH were input into the SCUNet model for image enhancement. Then, the ES images were used to train the GCT-DenseNet121 model. To achieve the optimal model, a validation set was utilized for the tuning of the model’s parameters, and an internal test set was employed for performance evaluation. Additionally, HQS and LQS images from three other hospitals were used as external datasets to assess the model’s generalizability. Ablation studies were also conducted to validate the effectiveness of the SCUNet and GCT.

### Generation of paired HQS and GLQS images for SCUNet model

**Fig. 9:**
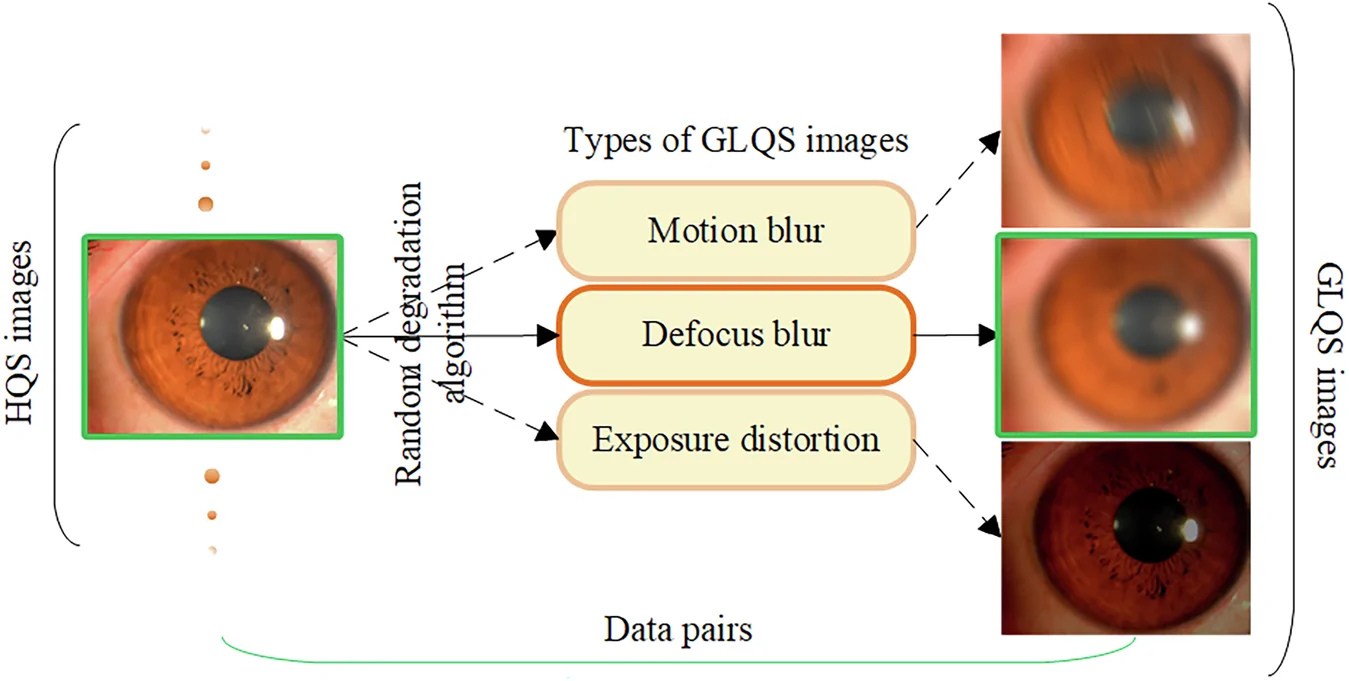
Generation of HQS and GLQS image pairs using a random degradation algorithm. HQS, high-quality slit-lamp. GLQS generated a low-quality slit-lamp.

To train the SCUNet model, it is essential to construct data pairs of HQS and GLQS images. In this study, an image degradation algorithm^52^ was used to simulate LQS images that might be encountered in clinical practice. Specifically, as illustrated in Fig. 9, a random image quality degradation algorithm was applied to HQS images to generate GLQS images, including MB, DB, and ED. Notably, both the type and degree of degradation algorithms were randomly selected. The GLQS images were then paired with the corresponding HQS images for training and validation of the SCUNet model. The formalized expressions of the degradation algorithm are presented in Eqs. 12–14.

The original image *I*(*x*,*y*) is convolved with either a motion-blur or defocus-blur kernel to simulate the respective degradation effects. Specifically, the MB image *I*_motion_(*x*,*y*) is generated by convolving *I*(*x*,*y*) with the spatially variant motion kernel *K*(*x*,*y*), as formally expressed in Eq. (12). Similarly, the DB image *I*_defocus_(*x*,*y*) is obtained by convolving *I*(*x*,*y*) with a defocus kernel, as defined in Eq. (13).

12 $${I}_{motion}(x,y)=I(x,y)* K(x,y)=\mathop{\sum }\limits_{i=-\infty }^{\infty }\mathop{\sum }\limits_{j=-\infty }^{\infty }I(i,j)\cdot K(x-i,y-j)$$

13 $${I}_{defocus}(x,y)=\frac{1}{2\pi {\sigma }^{2}}\mathop{\sum }\limits_{i=-\infty }^{\infty }\mathop{\sum }\limits_{j=-\infty }^{\infty }I(x,y)\cdot \exp (-\frac{{(x-i)}^{2}+{(y-j)}^{2}}{2{\sigma }^{2}})$$

where (*x*, *y*) denotes the spatial coordinate of the original image, (*i*, *j*) represents the discrete coordinate iterated over the convolution kernel, and σ indicates the standard deviation of the defocus blur.

Additionally, the ED image *I*_exposure_(*x*, *y*) is generated by randomly adjusting the luminance and contrast of each pixel, as described in Eq. (14).

14 $${I}_{\exp osure}(x,y)=\alpha \cdot I(x,y)+\beta$$

where *α* and *β* are the adjustment factors of contrast and luminance, respectively.

### SCUNet model for automatic enhancement of LQS images

The Swin-Conv (SC) module integrates the Swin Transformer (SwinT)^49^ and residual convolutional (RConv)^53^ blocks, facilitating efficient feature extraction and the seamless fusion of spatial information. In this study, the SC module is embedded into the U-Net architecture, and a novel Swin-Conv-U-Net (SCUNet) model is proposed. By leveraging the multiscale feature fusion capabilities of the SC module, SCUNet significantly enhances the quality of LQS images. The SCUNet model not only captures intricate details of keratitis lesions but also preserves feature consistency across different image resolutions. Trained on paired HQS and GLQS images, the SCUNet model learns the mapping relationship from GLQS to HQS images. Additionally, through the application of an adversarial training strategy, the ES images are generated from the trained SCUNet model, serving as input for the subsequent automatic diagnosis model of keratitis.

Figure 7a illustrates the architecture of the SCUNet model. The backbone of the SCUNet generator consists of four scales, each of which performs downsampling via 2 × 2 stride convolution (SConv) and upsampling via 2 × 2 transpose convolution (TConv). A residual connection is employed between SConv and TConv to maintain consistency in feature dimensions. The number of channels increases progressively from 64 at the first scale to 512 at the fourth scale.

In the SCUNet architecture, four SC modules are integrated for downsampling and upsampling operations at each scale. The SC module combines traditional convolutional operation with the non-local attention mechanism of the SwinT, enabling effective capture of global information and long-range dependencies within the input image. Specifically, the forward propagation process of each SC module is mathematically formulated in Eqs. (15, 16). Initially, the input feature map *X* is passed through a 1 × 1 convolution, which splits it into two components, *X*_1_ and *X*_2_. Subsequently, the feature maps *X*_1_ and *X*_2_ are separately processed through a SwinT block and an RConv block, outputting *Y*_1_ and *Y*_2_, respectively. Finally, the outputs *Y*_1_ and *Y*_2_ are concatenated and fused with the original input feature map *X* through a residual connection, yielding the final output *Z* of the SC module, as shown in Eq. (17).

15 $${X}_{1},{X}_{2}=Split(Conv1\times 1(X))$$

16 $${Y}_{1},{Y}_{2}=SwinT({X}_{1}),RConv{({\rm{X}}}_{2})$$

17 $$Z=Conv1\times 1(Concat({Y}_{1},{Y}_{2}))+X$$

SCUNet implements an adversarial training strategy to enhance the fidelity of generated images. This ensures that the texture and detail of the generated images closely resemble those of real images, rather than merely producing clearer but unrealistic ones. The discriminator (shown in Fig. 7a) is a deep CNN that extracts high-level features through a series of convolutional layers, batch normalization layers, and activation functions. The final feature map is passed through a sigmoid activation function, producing a probability score between 0 and 1 that indicates the likelihood that the generated image is real. The adversarial loss of the discriminator is formulated in Eq. (18).

18 $${L}_{D}(D)=-{E}_{x \sim {p}_{data}(x)}[\log (D(x))]-{E}_{z \sim {p}_{z}(z)}[\log (1-D(G(z)))]$$

where *x* and *G*(*z*) represent the real and generated images, respectively. The discriminator seeks to maximize log(*D*(*x*)), thereby driving the output for real images towards 1, while simultaneously maximizing log(1 − *D*(*G*(*z*))), which encourages the output for generated images to approach 0. By minimizing this loss function, the discriminator is trained to effectively differentiate between real and generated images.

The loss of the generator consists of three components: the adversarial loss (Eq. 19), pixel-level loss (Eq. 20), and structural similarity loss (Eq. 21). The total loss of the generator is the weighted sum of these three components, as illustrated in Eq. (22).

19 $${L}_{GAN}(G)={E}_{z \sim {p}_{z}(z)}[\log (1-D(G(z)))]$$

20 $${L}_{pixel}(G)={E}_{x,z}[{\Vert x-G(z)\Vert }_{2}^{2}]$$

21 $${L}_{SSIM}(G)=1-SSIM(x,G(z))$$

22 $${L}_{total}(G)={L}_{GAN}(G)+{\lambda }_{pixel}\cdot {L}_{pixel}(G)+{\delta }_{SSIM}\cdot {L}_{SSIM}(G)$$

where *λ*_pixel_ and *δ*_SSIM_ are weights that control the relative importance of each loss component. *L*_GAN_(*G*), *L*_pixel_(*G*), and *L*_SSIM_(*G*) represent the adversarial loss, pixel-level loss, and structural similarity loss of the generator, respectively.

### GCT-DenseNet121 model for keratitis diagnosis using ES images

In this study, by embedding the GCT module into the DenseNet121 network, an improved GCT-DenseNet121 was established for the automated diagnosis of keratitis, as shown in Fig. 7b. The application of multiple GCT modules aims to enhance the contextual information of the input feature maps by modeling the statistical properties of the data using a Gaussian distribution.

**Fig. 10:**
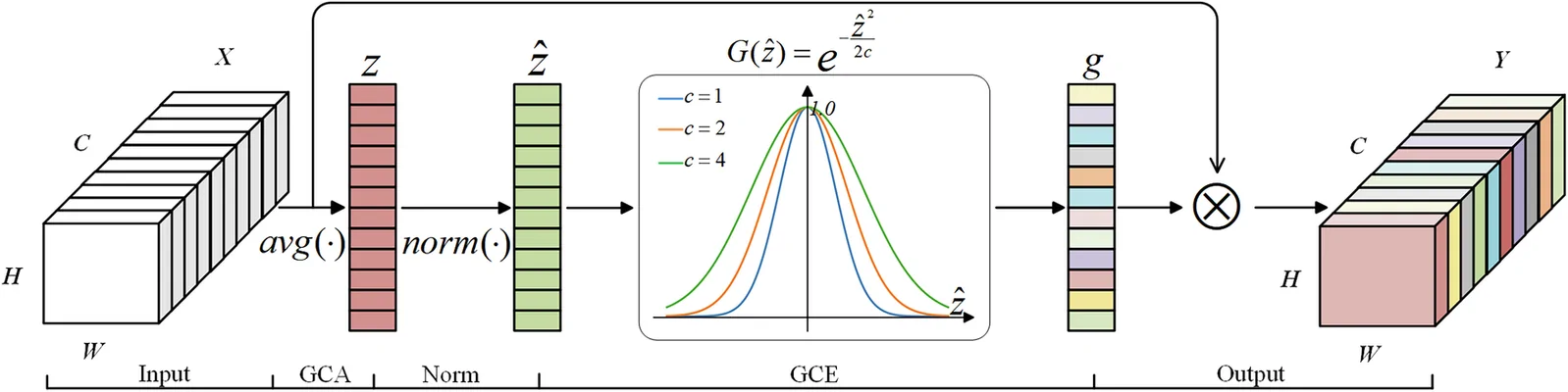
Schematic diagram of the unfolded GCT module. GCT Gaussian context transformer, GCA global context aggregation, GCE Gaussian context excitation, Norm normalization.

Compared to channel attention mechanisms such as squeeze-and-excitation (SE) and efficient channel attention (ECA)^54,54^, the GCT exhibits superior effectiveness and efficiency. The GCT consists of three primary operations: global context aggregation (GCA), normalization (Norm), and Gaussian context excitation (GCE), with implementation details provided in Fig. 10. Lesions and complications of keratitis often manifest as small, diffuse, and multifocal patterns, posing challenges for local feature extraction methods in capturing their interdependencies. To address this limitation, the GCA module within GCT leverages global average pooling to aggregate contextual information across the entire feature map, thereby extracting statistical representations across different channels. This technique enhances the ability of GCT-DenseNet121 to model long-range dependencies between lesions and complications in ES images, a crucial factor in the automated diagnosis of keratitis. The formal mathematical expression of GCA is presented in Eq. (23).

23 $$z=avg(X)=\left\{{z}_{k}=\frac{1}{H\times W}\mathop{\sum }\limits_{i=1}^{W}\mathop{\sum }\limits_{j=1}^{H}{X}_{k}(i,j):k\in \{1,\mathrm{..}.,C\}\right\}$$

Where *X* represents the input feature map with dimensions *C* × *H* × *W*.

The Norm operation is performed by introducing a mean shift (*z*-*μ*) and an instance-specific factor (*σ*) to model the negative correlation between the assumed contexts, with its formal expression provided in Eqs. (24–26). A larger mean deviation in the global context results in a lower attention activation.

24 $$\mu =\frac{1}{C}\mathop{\sum }\limits_{k=1}^{C}{z}_{k}$$

25 $$\sigma =\sqrt{\frac{1}{C}{\mathop{\sum }\limits_{k=1}^{C}({z}_{k}-\mu )}^{2}}+\varepsilon$$

26 $$\hat{z}=\frac{1}{\sigma }(z-\mu )$$

where *ε* is a small constant introduced to ensure the numerical stability of the computed $\hat{z}$.

The GCE leverages a Gaussian function to regulate context excitation, as expressed in Eq. (27). The learnable parameter *c* represents the standard deviation of the Gaussian function, which controls the variability of channel attention activation. A larger standard deviation indicates a reduced disparity in attention activation across different channels.

27 $$g=G(\hat{z})={e}^{-\frac{{\hat{z}}^{2}}{2{c}^{2}}}$$

Finally, the activated attention *g* is multiplied with the input features *X*, yielding the output features *Y*, as shown in Eq. (28). This design enhances the feature extraction capability of the GCT-DenseNet121 for LQS images, thereby benefiting the automatic diagnosis of keratitis.

28 $$Y={e}^{-\frac{norm{(avg(X))}^{2}}{2{c}^{2}}}X$$

### Ethical approval

This study was approved by the Ethics Review Committee of NEH (approval number 2021-xjx-009) and conducted in accordance with the principles of the Declaration of Helsinki. All slit-lamp images were anonymized prior to being transferred to research investigators, with patient identifiers such as names, ages, and medical histories removed. Informed consent was waived due to the retrospective nature of the data acquisition and the use of deidentified images.

## Supplementary information

Supplementary Material

## Data Availability

The data supporting the findings of this study are available within the article. The original hospital-sourced datasets are not publicly available due to restrictions required to protect patient confidentiality, as mandated by the National Health Commission of China and the participating institutions. These data may be made available for non-commercial academic research upon reasonable request to the corresponding author (Zhongwen Li), subject to institutional approval.

## Code availability

The code and example data used in this study are available on GitHub at https://github.com/jiangjiewei/AKDF-LQSI.
