# Supplementary material for: Automated diagnosis of keratitis from low-quality slit-lamp images using an improved generative adversarial network
Source: NPJ Digit Med. 2026 Jul 3;9:814. doi: 10.1038/s41746-026-02952-4 (PMC13696720; doi:10.1038/s41746-026-02952-4)
Supplement: Supplementary file 1 [file 41746_2026_2952_MOESM1_ESM.pdf]

Supplementary Information

Automated diagnosis of keratitis from low-quality slit-lamp images using an improved generative adversarial network

1. **Supplementary Figure 1. Comparison of PR curves between AKDF-LQSI and baseline architectures for keratitis diagnosis on the internal NEH and external LQS datasets. a** PR curves on the internal NEH LQS test set. **b** PR curves on the external LQS test set. PR, Precision–Recall. LQS, low-quality slit-lamp. AKDF-LQSI, automated keratitis diagnosis framework for LQS images. NEH, Ningbo Eye Hospital.

2. **Supplementary Figure 2. Interpretability analysis of AKDF-LQSI on correctly classified samples using SHAP.** Red regions highlight features that positively contribute to the prediction, suggesting a higher likelihood of a specific lesion or tissue, whereas blue regions highlight features that negatively contribute, indicating a lower likelihood. SHAP, SHapley Additive exPlanations. AKDF-LQSI, automated keratitis diagnosis framework for LQS images. Normal, normal cornea. Other, other cornea abnormalities.

3. **Supplementary Figure 3. Interpretability analysis of AKDF-LQSI on misclassified samples using Grad-CAM.** AKDF-LQSI, automated keratitis diagnosis framework for LQS images. Normal, normal cornea. Other, other cornea abnormalities. LQS, low-quality slit-lamp. ES, enhanced slit-lamp. Grad-CAM, Gradient-weighted Class Activation Mapping.

4. **Supplementary Figure 4. Interpretability analysis of AKDF-LQSI on misclassified samples using SHAP.** Red regions highlight features that positively contribute to the prediction, suggesting a higher likelihood of a specific lesion or tissue, whereas blue regions highlight features that negatively contribute, indicating a lower likelihood. SHAP,

SHapley Additive exPlanations. AKDF-LQSI, automated keratitis diagnosis framework for LQS images. Normal, normal cornea. Other, other cornea abnormalities.

5. **Supplementary Figure 5. Representative cases of discrepancies between AKDF-LQSI and clinician ophthalmologists in the contest dataset. a** Cases where AKDF-LQSI achieved correct diagnoses despite consensus errors by all four ophthalmologists. **a1** Normal cornea. **a2** Keratitis. **a3** Other cornea abnormalities. **b** Cases with all four ophthalmologists' consensus correct but AKDF-LQSI misdiagnoses. **b1** Keratitis. **b2** Normal cornea. **b3** Other cornea abnormalities.
6. **Supplementary Figure 6. Representative cases of slit-lamp images with predicted probability provided by AKDF-LQSI.** The predicted probability map for each category is displayed in the lower-right corner of the slit-lamp image. **a, d** Normal cornea. **b, e** Keratitis. **c, f** Other cornea abnormalities.
7. **Supplementary Figure 7.** Consistency comparisons between generated and real LQS images across keratitis, normal cornea, and other corneal abnormalities. **a** Pixel-level comparison of generated and real LQS images using histogram analysis. The average pixel-intensity profiles of generated LQS images closely align with those of multi-center real LQS images, indicating similar distributional patterns. **b** Feature-level consistency comparison using t-SNE visualization. High-level features extracted from randomly sampled 400 GLQS images and 400 real LQS images using a DenseNet121 pretrained on ImageNet were projected into a two-dimensional space. The resulting embeddings show closely intermixed clusters, suggesting no discernible separation between the two domains. LQS, low-quality slit-lamp. GLQS, generated low-quality slit-lamp. t-SNE, t-distributed stochastic neighbor embedding.
8. **Supplementary Table 1. Detailed hyperparameter configurations of the SCUNet and GCT-DenseNet121 models.**
9. **Supplementary Table 2. Computational efficiency of the SCUNet and GCT-DenseNet121 models.**

10. **Supplementary Table 3. Performance evaluation of SCUNet on three types of GLQS images in the external test set.**
11. **Supplementary Table 4. Comparative analysis of diagnostic discrepancies between AKDF-LQSI and clinician ophthalmologists in the contest dataset.**

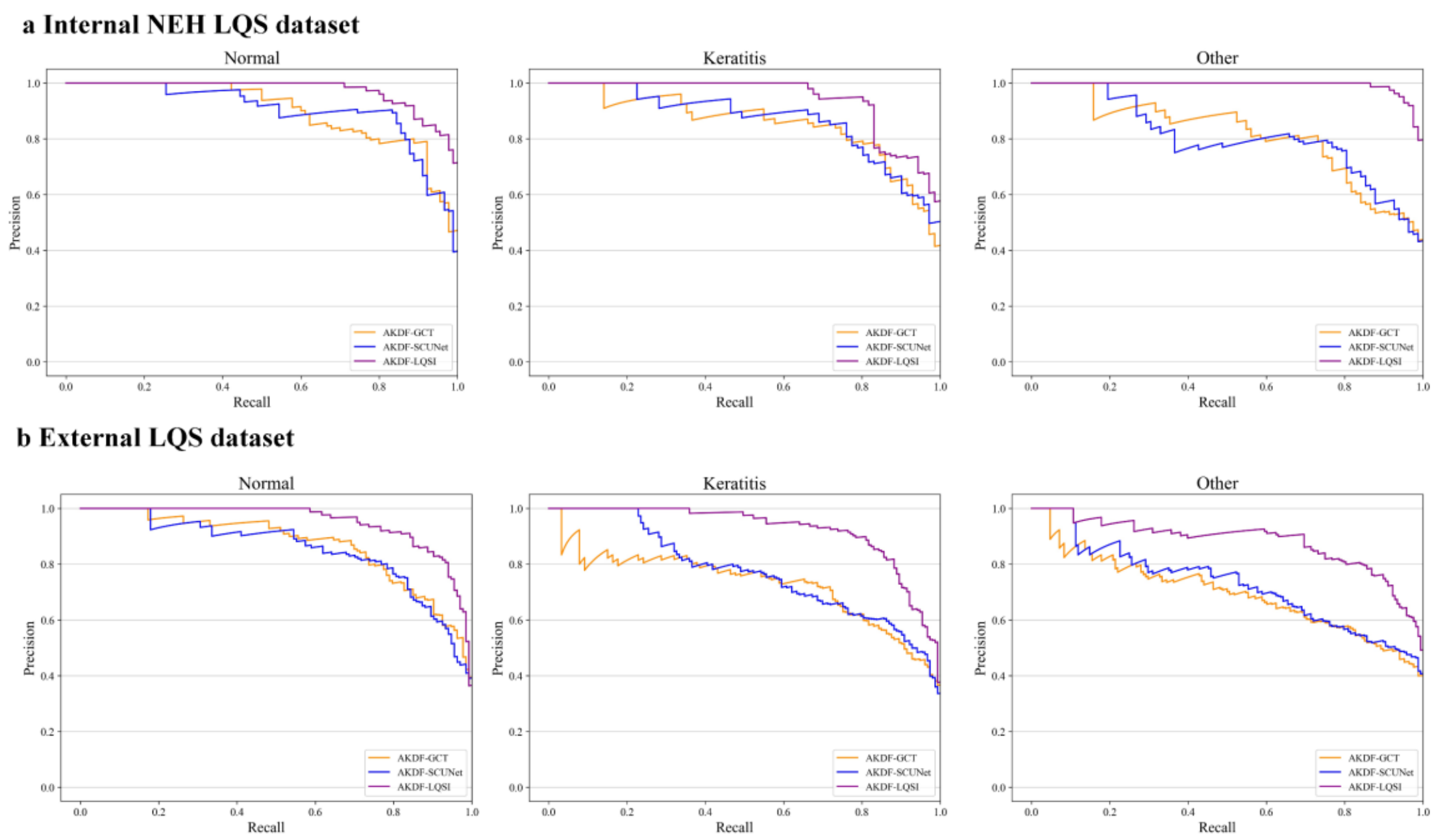

Supplementary Figure 1. Comparison of PR curves between AKDF-LQSI and baseline architectures for keratitis diagnosis on the internal NEH and external LQS datasets. a PR curves on the internal NEH LQS test set. **b** PR curves on the external LQS test set. PR, Precision–Recall. LQS, low-quality slit-lamp. AKDF-LQSI, automated keratitis diagnosis framework for LQS images. NEH, Ningbo Eye Hospital.

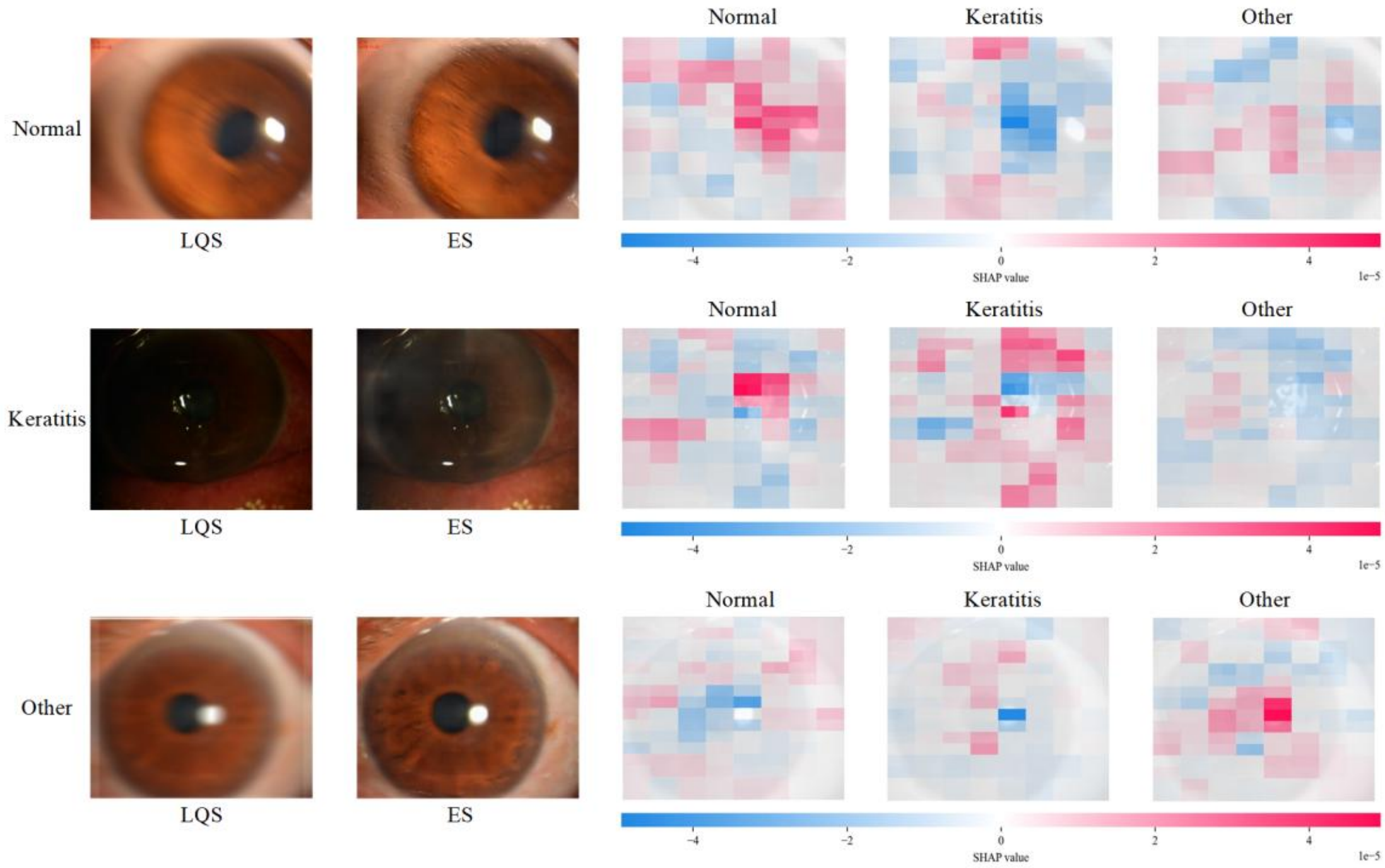

Supplementary Figure 2. Interpretability analysis of AKDF-LQSI on correctly classified samples using SHAP. Red regions highlight features that positively contribute to the prediction, suggesting a higher likelihood of a specific lesion or tissue, whereas blue regions highlight features that negatively contribute, indicating a lower likelihood. SHAP, SHapley Additive exPlanations. AKDF-LQSI, automated keratitis diagnosis framework for LQS images. Normal, normal cornea. Other, other cornea abnormalities. LQS, low-quality slit-lamp. ES, enhanced slit-lamp.

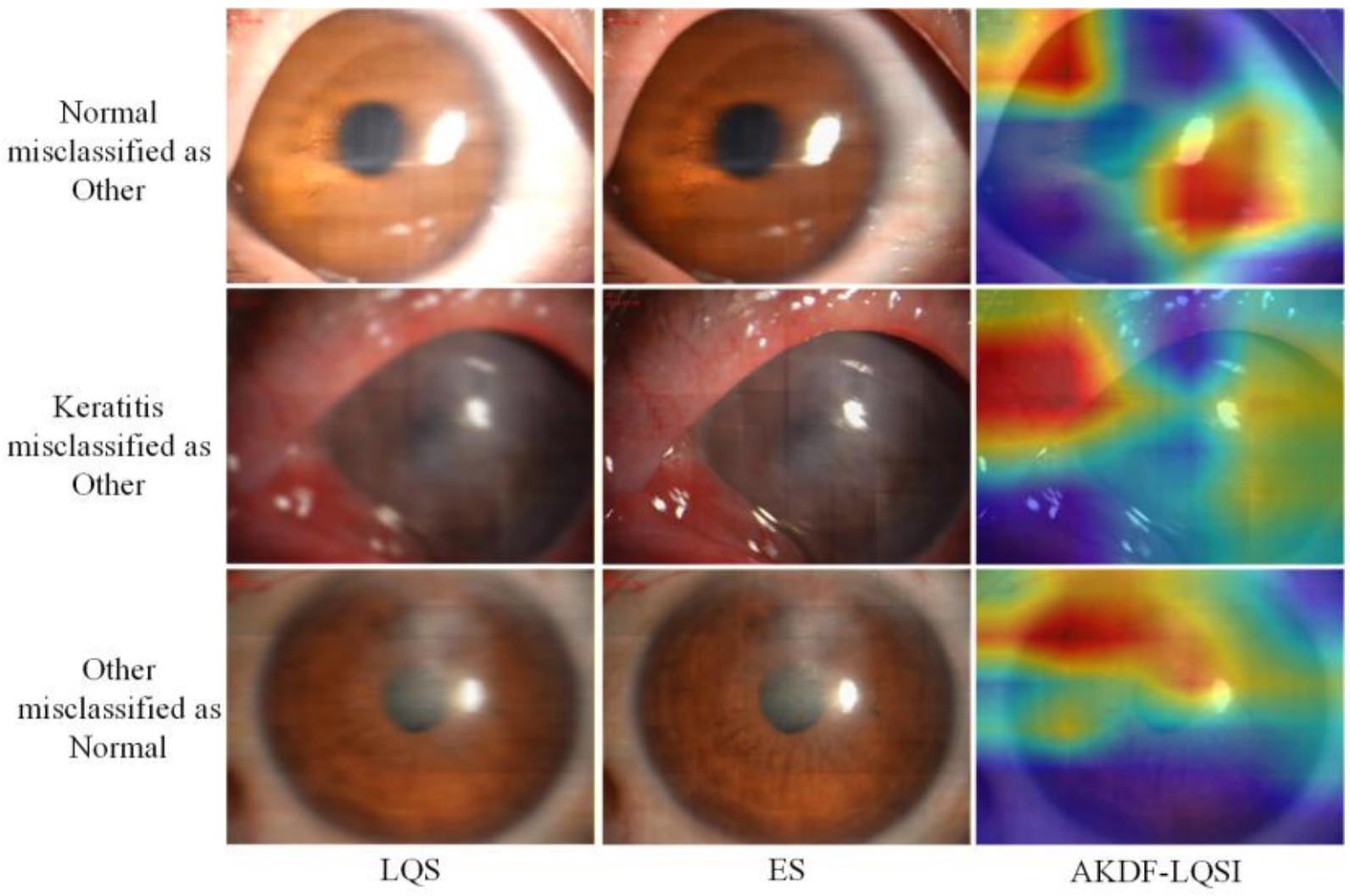

Supplementary Figure 3. Interpretability analysis of AKDF-LQSI on misclassified samples using Grad-CAM. AKDF-LQSI, automated keratitis diagnosis framework for LQS images. Normal, normal cornea. Other, other cornea abnormalities. LQS, low-quality slit-lamp. ES, enhanced slit-lamp. Grad-CAM, Gradient-weighted Class Activation Mapping.

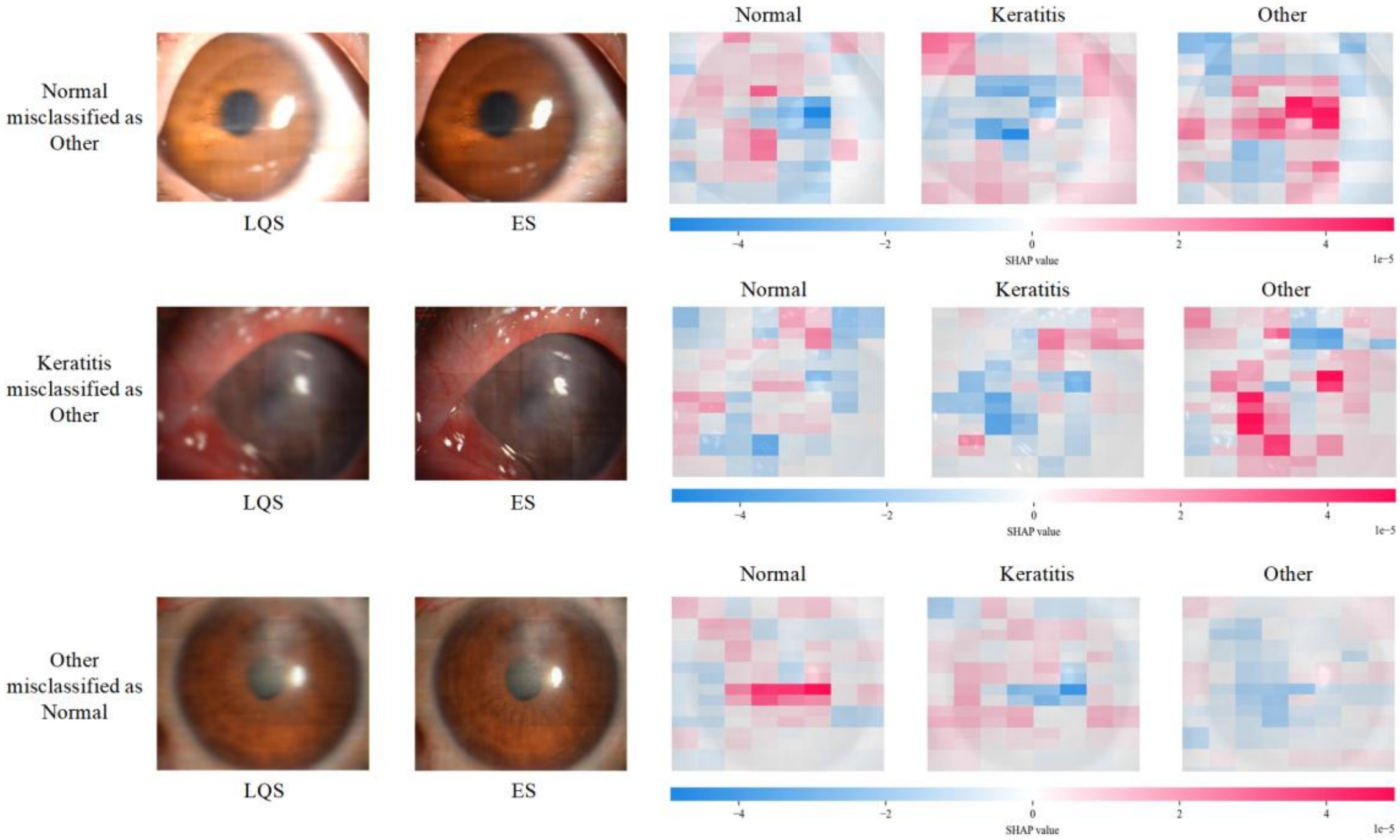

Supplementary Figure 4. Interpretability analysis of AKDF-LQSI on misclassified samples using SHAP. Red regions highlight features that positively contribute to the prediction, suggesting a higher likelihood of a specific lesion or tissue, whereas blue regions highlight features that negatively contribute, indicating a lower likelihood. SHAP, SHapley Additive exPlanations. AKDF-LQSI, automated keratitis diagnosis framework for LQS images. Normal, normal cornea. Other, other cornea abnormalities.

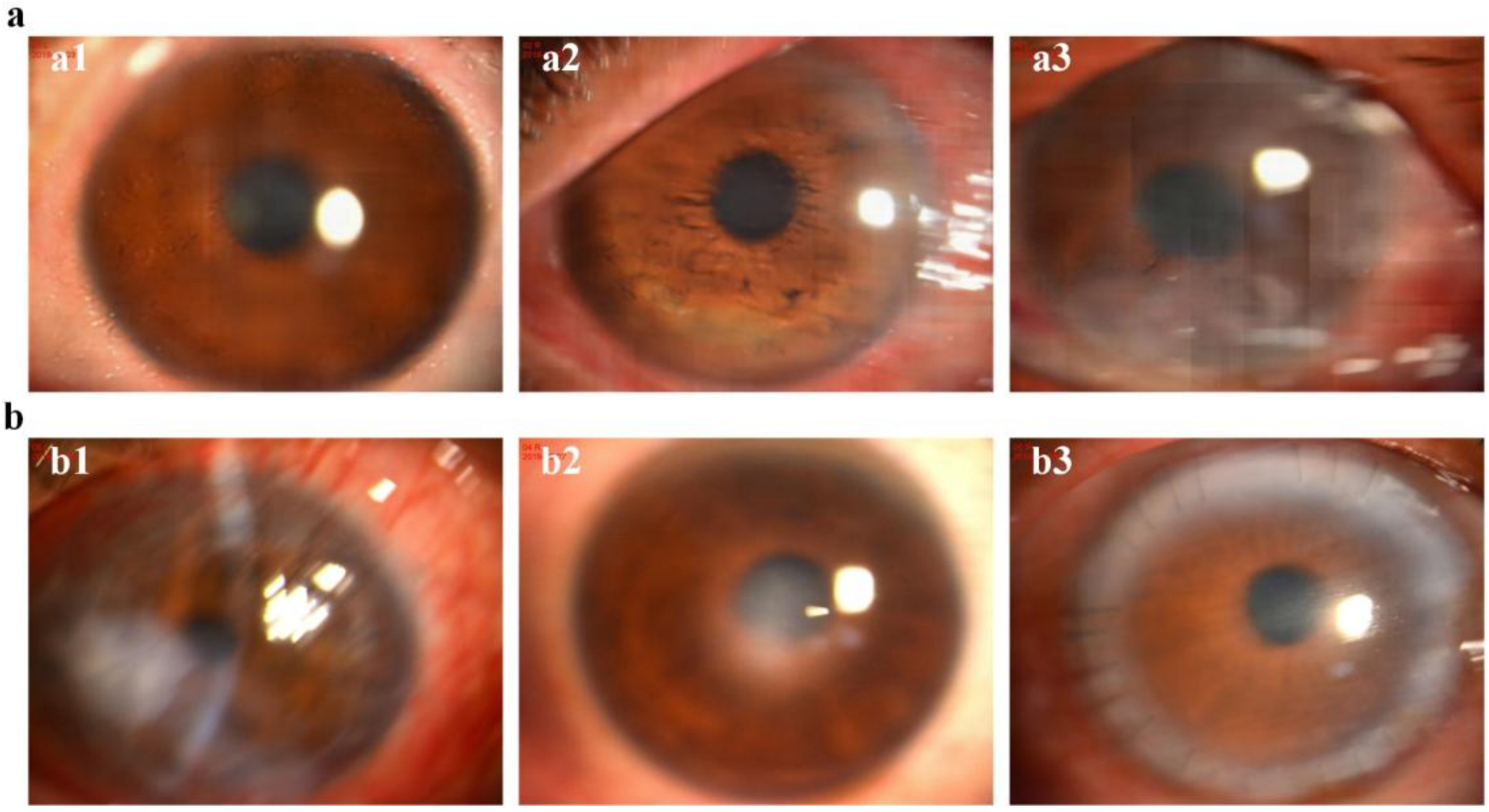

Supplementary Figure 5. Representative cases of discrepancies between AKDF-LQSI and clinician ophthalmologists in the contest dataset. a Cases where AKDF-LQSI achieved correct diagnoses despite consensus errors by all four ophthalmologists. **a1** Normal cornea. **a2** Keratitis. **a3** Other cornea abnormalities. **b** Cases with all four ophthalmologists' consensus correct but AKDF-LQSI misdiagnoses. **b1** Keratitis. **b2** Normal cornea. **b3** Other cornea abnormalities.

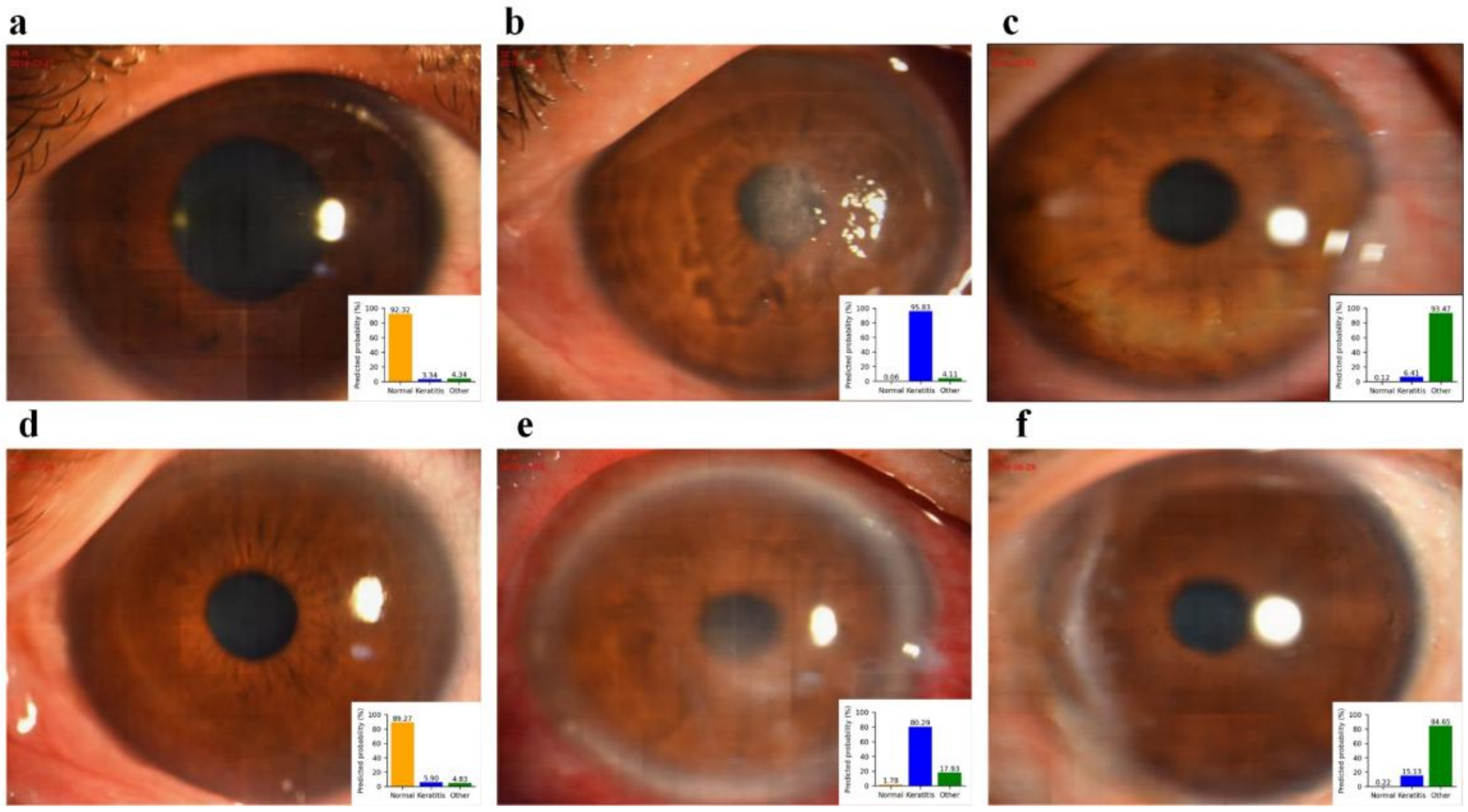

Supplementary Figure 6. Representative cases of slit-lamp images with predicted probability provided by AKDF-LQSI. The predicted probability map for each category is displayed in the lower-right corner of the slit-lamp image. **a, d** Normal cornea. **b, e** Keratitis. **c, f** Other cornea abnormalities.

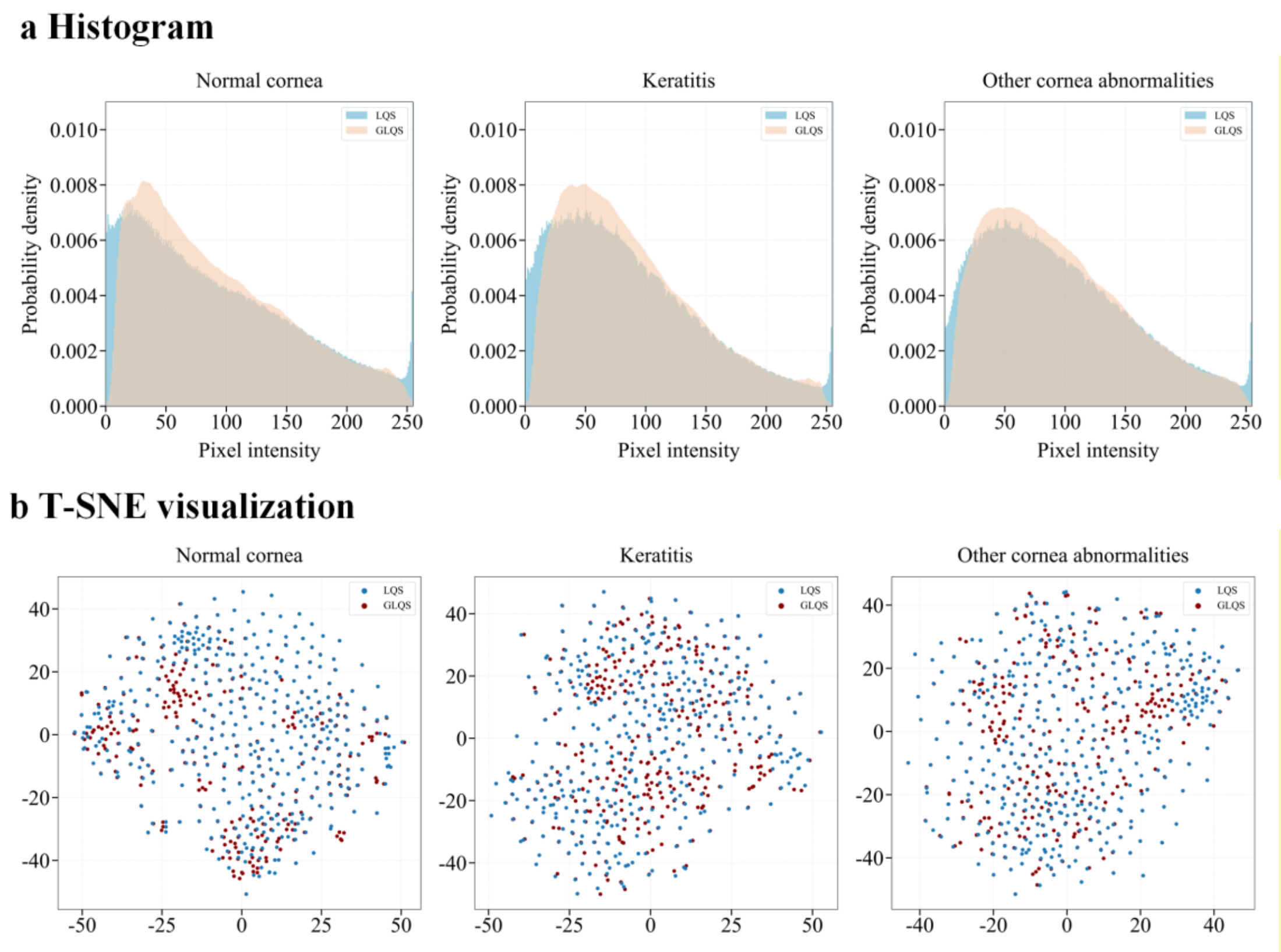

Supplementary Figure 7. Consistency comparisons between generated and real LQS images across keratitis, normal cornea, and other corneal abnormalities. a Pixel-level comparison of generated and real LQS images using histogram analysis. The average pixel-intensity profiles of generated LQS images closely align with those of multi-center real LQS images, indicating similar distributional patterns. **b** Feature-level consistency comparison using t-SNE visualization. High-level features extracted from randomly sampled 400 GLQS images and 400 real LQS images using a DenseNet121 pretrained on ImageNet were projected into a two-dimensional space. The resulting embeddings show closely intermixed clusters, suggesting no discernible separation between the two domains. LQS, low-quality slit-lamp. GLQS, generated low-quality slit-lamp. t-SNE, t-distributed stochastic neighbor embedding.

Supplementary Table 1. Detailed hyperparameter configurations of the SCUNet and GCT-DenseNet121 models.

Item	SCUNet	GCT-DenseNet121
Deep learning framework	PyTorch	PyTorch
GPU configuration	4×NVIDIA TITAN RTX	NVIDIA TITAN RTX
Training epochs	120	80
Batch size	4	64
Optimizer	Adam	Adam
Initial learning rate	3e-4	0.005
β_1 (Adam)	0.5	0.9
β_2 (Adam)	0.999	0.999
Weight decay	-	5e-5
Model initialization	Random initialization	ImageNet pre-trained weights
Input image size	512×512 pixels	224×224 pixels

Supplementary Table 2. Computational efficiency of the SCUNet and GCT-DenseNet121 models.

Model	Size	Trainable parameters	Training time	Testing time
SCUNet	36.9MB	1.5e + 07	99.4h	0.068s
GCT-DenseNet121	54.2MB	7.1e + 06	5.4h	0.025s

Footnote: Training time refers to the total duration required to train each model. Testing time represents the average inference time per image. MB, Mbyte.

Supplementary Table 3. Performance evaluation of SCUNet on three types of GLQS images in the external test set.

Metrics	GLQS images			ES images		
	MB	DB	ED	MB	DB	ED
Mean	96.93	102.56	106.43	92.21	95.38	96.16
SD	60.89	65.36	69.11	56.95	58.52	58.04
AG	12.29	18.36	28.65	18.51	21.16	25.31
MSE	79.81	59.31	93.11	67.80	41.93	72.76
PSNR	19.37	24.59	19.77	19.38	29.40	25.07
SSIM	0.65	0.75	0.76	0.70	0.84	0.94

Footnote: GLQS, generated low-quality slit-lamp. ES, enhanced slit-lamp. MB, motion blur. DB, defocus blur. ED, exposure distortion. SD, standard deviation. AG, average gradient. MSE, mean squared error. PSNR, peak signal-to-noise ratio. SSIM, structural similarity index.

Supplementary Table 4. Comparative analysis of diagnostic discrepancies between AKDF-LQSI and clinician ophthalmologists in the contest dataset.

Diagnostic Categories	AKDF-LQSI Correct / All Ophthalmologists Incorrect	All Ophthalmologists Correct / AKDF-LQSI Incorrect
Normal	1	1
Keratitis	2	2
Other	3	2

Footnote: Normal, normal cornea. Other, other cornea abnormalities.
